# Application of the Triazolization Reaction to Afford Dihydroartemisinin Derivatives with Anti-HIV Activity

**DOI:** 10.3390/molecules22020303

**Published:** 2017-02-17

**Authors:** Sampad Jana, Shabina Iram, Joice Thomas, Muhammad Qasim Hayat, Christophe Pannecouque, Wim Dehaen

**Affiliations:** 1 Molecular Design and Synthesis, Department of Chemistry, KU Leuven, Celestijnenlaan 200F, 3001 Leuven, Belgium; sampad.jana@kuleuven.be (S.J.); joicetk2003@gmail.com (J.T.); 2 Department of Plant Biotechnology, Atta-Ur-Rahman School of Applied Biosciences (ASAB), National University of Sciences and Technology (NUST), H-12 Islamabad, Pakistan; shabinairam@hotmail.com (S.I.); mqasimhayat@hotmail.com (M.Q.H.); 3 Department of Microbiology and Immunology, Laboratory of Virology and Chemotherapy, Rega Institute for Medical Research, KU Leuven, Herestraat 49, B-3000 Leuven, Belgium

**Keywords:** triazole, artemisinin, triazolization, organocatalytic, multicomponent reaction

## Abstract

Artemisinin and synthetic derivatives of dihydroartemisinin are known to possess various biological activities. Post-functionalization of dihydroartemisinin with triazole heterocycles has been proven to lead to enhanced therapeutic potential. By using our newly developed triazolization strategy, a library of unexplored fused and 1,5-disubstituted 1,2,3-triazole derivatives of dihydroartemisinin were synthesized in a single step. All these newly synthesized compounds were characterized and evaluated for their anti-HIV (Human Immunodeficiency Virus) potential in MT-4 cells. Interestingly; three of the synthesized triazole derivatives of dihydroartemisinin showed activities with half maximal inhibitory concentration (IC_50_) values ranging from 1.34 to 2.65 µM.

## 1. Introduction

**Figure 1:**
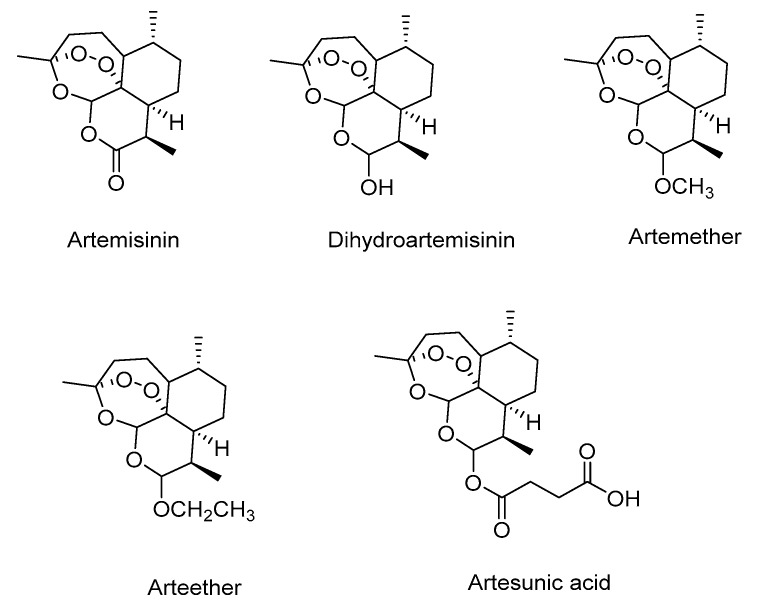
Structures of artemisinin and its semi-synthetic derivatives.

The human immunodeficiency virus (HIV), the causative agent of the acquired immunodeficiency syndrome (AIDS), has been plaguing the human race for more than thirty years [1]. According to the global statistics of UNAIDS, it has been estimated that globally 36.7 million people were suffering from HIV/AIDS in 2015 [2]. Even though more than thirty drugs targeting different steps of the viral life cycle are either approved or in clinical stages to treat HIV/AIDS [2], a cure remains elusive. Moreover, emergence of HIV strains that are no longer sensitive to the drug cocktails employed is leading to inability to completely block the viral replication [3]. Thus, finding new anti-HIV agents that are less toxic and more effective in targeting HIV reservoirs in the body is still needed. Artemisinin, a naturally occurring 1,2,4-trioxane sesquiterpene, first isolated in 1972 from a Chinese medicinal plant (*Artemisia annua* L.) that had been used as a treatment for fever for many centuries [4]. Interestingly, artemisinin has been used successfully at nanomolar concentrations against both chloroquine-sensitive and -resistant strains of *Plasmodium falciparum*. However, its therapeutic efficiency is not optimal due to poor oral bioavailability and limited solubility [5,6]. Reduction of the carbonyl group of artemisinin leads to the synthesis of dihydroartemisinin in high yields without disrupting the unusual peroxide linkage of artemisinin which has in turn lead to the development of a series of semi-synthetic first generation derivatives including the oil soluble artemether and arteether, and water soluble sodium artesunate (Figure 1) [5]. Derivatives of artemisinin have shown improved potency as antimalarial and anticancer agent as compared to artemisinin itself [5,7].

Dihydroartemisinin has become a subject of considerable interest in medicinal chemistry for its therapeutic value, better solubility, and being a versatile precursor for the synthesis of wide variety of other artemisinin derivatives [4]. Furthermore, derivatives of dihydroartemisinin are found to have antiproliferative, antibacterial, antiviral, and immunosuppressive activities [8,9,10,11,12,13,14,15,16,17,18,19,20,21]. Even though it displays a high activity profile, dihydroartemisinin does suffer some drawbacks such as neurotoxicity in animal models, short pharmacological half-life, and recrudescence of the disease [22,23]. The shortcomings of the current artemisinin derivatives and emergence of multidrug resistance for artemisinin based combination therapies [24] are the motivation to search for novel artemisinin compounds.

Although 1,2,3-triazole derivatives of artemisinin have been synthesized and evaluated for biological activity [25,26], no attempt has been made to synthesize 1,5-disubstituted 1,2,3-triazole derivatives of dihydroartemisinin in a single step and metal-free conditions. The widespread applications of 1,2,3-triazoles have triggered the demand for metal free and easier access to this privileged scaffold. Herein we disclose the implementation of our recently developed triazolization strategy [27,28,29] in which dihydroartemisinin scaffold was joined with variety of interesting heterocyclic moieties. We presume that this strategy could help in synthesizing a series of compound with minimal effort to obtain various fused and 1,5-disubstituted triazole derivatives of dihydroartemisinin.

## 2. Results and Discussion

### 2.1. Organocatalytic Synthesis of Triazole Functionalized Artemisinin

As an effective method of functionalizing dihydroartemisinin with triazole heterocycle, we implemented our recently developed triazolization strategy [27,28,29]. The method generally involves the reaction of a primary amine and an enolizable ketone **6** in the presence of 4-nitrophenyl azide **7** as a source of dinitrogen. The mechanistic study of this reaction indicated that an equilibrium exists between imine and enamine followed by an enamine mediated [3+2] cycloaddition reaction. This leads to the formation of a triazole intermediate followed by elimination of nitroaniline which gave the fused or 1,5-disubstituted 1,2,3-triazole derivative of dihydroartemisinin.

**Scheme 1:**
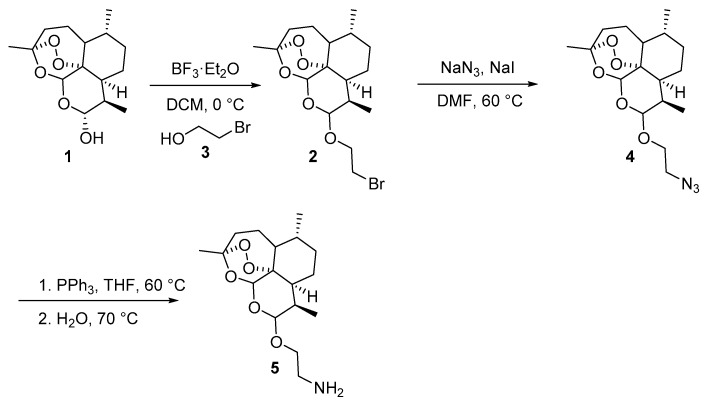
Synthetic route towards the preparation of amine precursor **4**. DCM: dichloromethane; DMF: dimethylformamide; THF: tetrahydrofuran.

Initially, the amine functionalized dihydroartemisinin **5** was synthesized by a previously reported procedure [30]. It involves the conversion of dihydroartemisinin into 10-bromoethoxydihydroartemisinin (**2**) by using 2-bromoethanol (**3**) in the presence of BF_3_·Et_2_O as a catalyst. Reaction of sodium azide with 10-bromoethoxydihydroartemisinin (**2**) afforded 2-(10β-dihydroartemisinoxy) ethyl azide (**4**) in 95% isolated yield (Scheme 1). The azido compound was then reduced to 2-(10β-dihydroartemisinoxy) ethyl amine (**5**) in 74% yield via a Staudinger reduction (Scheme 1).

The amine-modified dihydroartemisinin was then tested for the triazolization strategy with various aromatic and enolizable ketones. Cyclic ketones such as tetralones and their derivatives reacted smoothly under the modified triazolization conditions leading to the expected product in moderate yield. Acetophenone was also converted to the desired product **8f** (Table 1). Next, the versatility of this triazolization reaction was shown by functionalizing the modified dihydroartemisinin **5** with the male sex hormone analogue dihydrotestosterone and the triterpene betulonic acid giving rise to the fused triazole derivatives **8k** and **8l**, respectively, in moderate yield shown in Table 1.

**Scheme 2:**
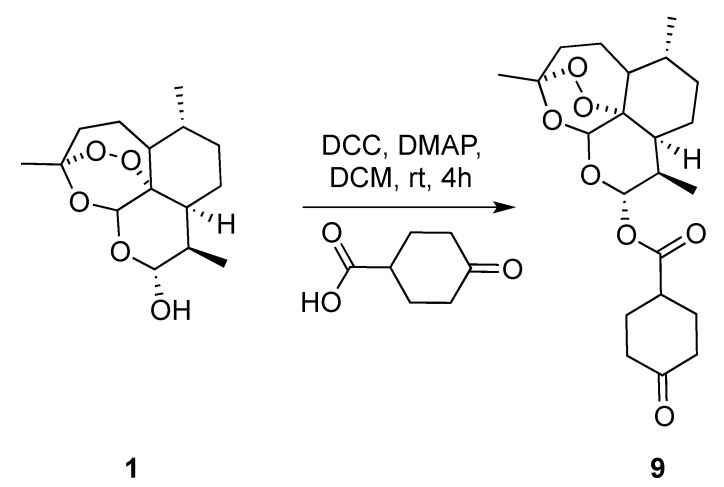
Synthetic route towards the preparation of keto precursor **9**. DCC: *N*,*N*’-dicyclohexylcarbodiimide; DMAP: 4-dimethylaminopyridine

In the next series of experiments, we reversed the strategy by functionalizing dihydroartemisinin scaffold with symmetrical enolizable cyclic ketone. The building block **9** was obtained by classical DCC (*N*,*N*’-dicyclohexylcarbodiimide) coupling reaction of dihydroartemisinin with cyclohexanone-4-carboxylic acid isolated in 82% yield (Scheme 2).

The versatility of our triazolization methodology was further exploited by condensing with various commercial and biologically relevant primary amine derivatives. Ketone-modified dihydroartemisinin **9** was treated with various benzylamines which led to the synthesis of triazole conjugate in good yield (Table 2). Tryptamine also works fine under these reaction circumstances leading to the expected product **11d** in moderate yield (Table 2).

### 2.2. Anti-Human Immunodeficiency Virus Activity Evaluation

The newly synthesized dihydroartemisinin triazole derivatives were evaluated for their inhibitory effects (IC_50_) on the replication of wild-type HIV-1 (III_B_) and HIV-2 (ROD) in MT-4 cell cultures, in parallel with their cytotoxicities (CC_50_) using the 3-(4,5-dimethylthiazol-2-yl)-2,5-diphenyl tetrazolium bromide (MTT) method [31]. A series of nucleoside reverse transcriptase inhibitors (NRTIs)—i.e., azidothymidine (AZT), lamivudine (3TC) and didanosine (DDI)—and non-nucleoside reverse transcriptase inhibitors (NNRTIs)—i.e., nevirapine (NVP), efavirenz (EFV), and etravirine (ETR)—were included as reference compounds. The biological results are represented as IC_50_ and CC_50_ values in Table 3.

Most of the new triazole analogues of dihydroartemisinin are completely devoid of anti-HIV activity. However, the unsubstituted β-2-tetralone analogue **8b** exhibited moderate activity against wild-type (wt) HIV-1 III_B_ with IC_50_ value of 2.78 μM, whereas its α-tetralone analogue **8a** did not show any activity. Both the mono and di-methoxy β-tetralone analogues **8e** and **8c**, are slightly less active against HIV-1 wt IC_50_ values of 5.18 and 4.06 μM respectively. The active molecules were also evaluated for their inhibitory activity against a double reverse transcriptase mutant (K103N; Y181C) HIV-1 strain (RES056). The derivatives **8b**, **8c** and **8e**, just like the first generation NNRTI nevirapine, completely lose their inhibitory activity, whereas the anti-HIV activity of efavirenz and etravirine is only slightly affected. Therefore, the active β-tetralone derivatives most probably act via an NNRTI-type mode of action.

## 3. Experimental Section

### 3.1. General Information

^1^H- and ^13^C-NMR spectra were recorded on commercial instruments (Bruker AMX 400 MHz or Bruker Avance II^+^ 600 MHz, Bruker, Rheinstetten, Germany) and chemical shifts (δ) are referenced to tetramethylsilane (^1^H), or the internal (NMR) solvent signal (^13^C). For column chromatography 70–230 mesh silica 60 (E. M. Merck, Steinheim am Albuch, Germany) was used as the stationary phase. Chemicals received from commercial sources were used without further purification. Synthesis of **5** was carried out based on the literature report and exactly matched with reported NMR data [30]. Other products’ NMR spectra see Supplementary Materials.

### 3.2. General Procedure for Modified Triazolization Reaction

A flame-dried screw-capped reaction tube equipped with magnetic stirring bar was charged with amine, ketone, 4-nitrophenylazide, and 4 Å molecular sieves. The mixture was dissolved in toluene (0.4 mL) and stirred at 60 °C for 18–36 h. The reaction was monitored by Thin-layer chromatography and after completion of reaction, the solvent was removed in vacuo. The crude reaction mixture was then subsequently purified by column chromatography (silica gel) first using DCM (dichloromethane) as eluent to remove all 4-nitroaniline formed during the reaction, followed by a mixture of heptane and ethyl acetate as the eluent to afford the title product.

### 3.3. Characterization of the Products

*1-(2-(((3R,6R,8aS,9R,10R,12R,12aR)-3,6,9-Trimethyldecahydro-12H-3,12-epoxy[1,2]dioxepino[4,3-i]isochromen-10-yl)oxy)ethyl)-4,5-dihydro-1H-naphtho[1,2-d][1,2,3]triazole* (**8a**): **5** (60 mg, 0.183 mmol), α-tetralone (26.8 mg, 0.183 mmol), 4-nitrophenyl azide (36.1 mg, 0.220 mmol). Reaction time was 24 h. The product was purified by flash column chromatography (first DCM, followed by EtOAc/heptane = 3:2) to afford **8a** (42 mg, 48%) as an off-white semisolid: ^1^H-NMR (400 MHz, CDCl_3_) δ 7.94 (d, *J* = 7.4 Hz, 1H), 7.30 (td, *J* = 7.4, 1.8 Hz, 1H), 7.24–7.17 (m, 2H), 5.38 (s, 1H), 4.62 (d, *J* = 8.0 Hz, 1H), 4.60–4.54 (m, 1H), 4.53–4.44 (m, 1H), 4.23 (dt, *J* = 10.6, 4.3 Hz, 1H), 3.91 (ddd, *J* = 10.7, 8.6, 4.0 Hz, 1H), 3.09–2.91 (m, 4H), 1.87–1.81 (m, 1H), 1.71–1.62 (m, 3H), 1.60–1.49 (m, 3H), 1.47 (s, 3H), 1.44–1.35 (m, 1H), 1.29 (dt, *J* = 14.4, 3.2 Hz, 1H), 1.22–1.09 (m, 2H), 1.06–0.95 (m, 1H), 0.88 (dd, *J* = 6.3, 3.6 Hz, 6H). ^13^C-NMR (101 MHz, CDCl_3_) δ 143.34, 134.03, 133.69, 129.04, 128.19, 127.37, 127.35, 122.16, 107.27, 101.43, 97.25, 82.73, 68.54, 48.50, 45.24, 44.11, 39.98, 35.27, 34.55, 34.52, 32.81, 28.81, 23.87, 22.17, 19.41, 19.34, 18.76.

*3-(2-(((3R,6R,8aS,9R,10R,12R,12aR)-3,6,9-Trimethyldecahydro-12H-3,12-epoxy[1,2]dioxepino[4,3-i]isochromen-10-yl)oxy)ethyl)-4,5-dihydro-3H-naphtho[1,2-d][1,2,3]triazole* (**8b**): **5** (60 mg, 0.183 mmol), β-tetralone (26.8 mg, 0.183 mmol), 4-nitrophenyl azide (36.1 mg, 0.220 mmol). Reaction time was 16 h. The product was purified by flash column chromatography (first DCM, then EtOAc/heptane = 3:2) to afford **8b** (62 mg, 70%) as an off-white solid: m.p. 104–105 °C; ^1^H-NMR (400 MHz, CDCl_3_) δ 7.94 (d, *J* = 7.4 Hz, 1H), 7.30 (td, *J* = 7.4, 1.8 Hz, 1H), 7.24–7.17 (m, 2H), 5.38 (s, 1H), 4.62 (d, *J* = 8.0 Hz, 1H), 4.60–4.54 (m, 1H), 4.53–4.44 (m, 1H), 4.23 (dt, *J* = 10.6, 4.3 Hz, 1H), 3.91 (ddd, *J* = 10.7, 8.6, 4.0 Hz, 1H), 3.09–2.91 (m, 4H), 1.87–1.81 (m, 1H), 1.71–1.62 (m, 3H), 1.60–1.49 (m, 3H), 1.47 (s, 3H), 1.44–1.35 (m, 1H), 1.29 (dt, *J* = 14.4, 3.2 Hz, 1H), 1.22–1.09 (m, 2H), 1.06–0.95 (m, 1H), 0.88 (dd, *J* = 6.3, 3.6 Hz, 6H). ^13^C-NMR (101 MHz, CDCl_3_) δ 143.34, 134.03, 133.69, 129.04, 128.19, 127.37, 127.35, 122.16, 107.27, 101.43, 97.25, 82.73, 68.54, 48.50, 45.24, 44.11, 39.98, 35.27, 34.55, 34.52, 32.81, 28.81, 23.87, 22.17, 19.41, 19.34, 18.76.

*7,8-Dimethoxy-3-(2-(((3R,6R,8aS,9R,10R,12R,12aR)-3,6,9-trimethyldecahydro-12H-3,12-epoxy[1,2]dioxepino[4,3-i]isochromen-10-yl)oxy)ethyl)-4,5-dihydro-3H-naphtho[1,2-d][1,2,3]triazole* (**8c**): 5 (60 mg, 0.183 mmol), 6,7-dimethoxy-3,4-dihydronaphthalen-2(1*H*)-one (37.8 mg, 0.183 mmol), 4-nitrophenyl azide (36.1 mg, 0.220 mmol). Reaction time was 16 h. The product was purified by flash column chromatography (first DCM, followed by EtOAc/heptane = 3:2) to give 8c (67 mg, 67%) as an off-white semisolid: ^1^H-NMR (400 MHz, CDCl_3_) δ 7.49 (s, 1H), 6.76 (s, 1H), 5.38 (s, 1H), 4.62 (d, *J* = 7.9 Hz, 1H), 4.59–4.53 (m, 1H), 4.51–4.44 (m, 1H), 4.26–4.18 (m, 1H), 3.95 (s, 3H), 3.89 (s, 4H), 3.05–2.84 (m, 4H), 1.88–1.79 (m, 1H), 1.71–1.62 (m, 3H), 1.60–1.50 (m, 3H), 1.47 (s, 3H), 1.44–1.35 (m, 1H), 1.33–1.23 (m, 2H), 1.21–1.10 (m, 1H), 1.08–0.95 (m, 1H), 0.91–0.85 (m, 6H). ^13^C-NMR (101 MHz, CDCl_3_) δ 148.40, 148.20, 143.46, 133.09, 125.92, 121.85, 111.89, 107.25, 105.67, 101.41, 97.24, 82.71, 68.50, 56.26, 56.18, 48.52, 45.23, 44.12, 39.97, 35.26, 34.55, 34.51, 32.80, 28.45, 23.85, 22.16, 19.62, 19.32, 18.74.

*8-Nitro-3-(2-(((3R,6R,8aS,9R,10R,12R,12aR)-3,6,9-trimethyldecahydro-12H-3,12-epoxy[1,2]dioxepino[4,3-i]isochromen-10-yl)oxy)ethyl)-4,5-dihydro-3H-naphtho[1,2-d][1,2,3]triazole* (**8d**): **5** (60 mg, 0.183 mmol), 7-nitro-3,4-dihydronaphthalen-2(1*H*)-one (35 mg, 0.183 mmol), 4-nitrophenyl azide (36.1 mg, 0.220 mmol). Reaction time was 16 h. Flash column chromatography (first DCM, followed by EtOAc/heptane = 3:2) afforded **8d** (70.4 mg, 73%) as an off-white semisolid: ^1^H-NMR (400 MHz, CDCl_3_) δ 8.78 (d, *J* = 2.2 Hz, 1H), 8.12 (dd, *J* = 8.3, 2.3 Hz, 1H), 7.48 (d, *J* = 8.4 Hz, 1H), 5.38 (s, 1H), 4.91–4.87 (m, 2H), 4.61 (d, *J* = 7.9 Hz, 1H), 4.45–4.39 (m, 1H), 4.15–4.01 (m, 1H), 3.15–3.02 (m, 4H), 1.86–1.79 (m, 1H), 1.69–1.61 (m, 3H), 1.55–1.48 (m, 3H), 1.44 (s, 3H), 1.36–1.32 (m, 1H), 1.32–1.27 (m, 2H), 1.21–1.11 (m, 1H), 1.02–0.92 (m, 1H), 0.88–0.76 (m, 6H). ^13^C-NMR (101 MHz, CDCl_3_) δ 147.43, 145.81, 144.66, 131.02, 129.79, 126.95, 123.16, 118.31, 107.19, 101.28, 97.24, 82.76, 77.16, 68.37, 50.36, 45.28, 44.08, 39.56, 35.21, 34.58, 34.51, 32.61, 30.78, 29.85, 23.85, 22.17, 20.38, 19.18, 18.80.

*8-Methoxy-3-(2-(((3R,6R,8aS,9R,10R,12R,12aR)-3,6,9-trimethyldecahydro-12H-3,12-epoxy[1,2]dioxepino[4,3-i]isochromen-10-yl)oxy)ethyl)-4,5-dihydro-3H-naphtho[1,2-d][1,2,3]triazole* (**8e**): **5** (60 mg, 0.183 mmol), 7-methoxy-3,4-dihydronaphthalen-2(1*H*)-one (32.3 mg, 0.183 mmol), 4-nitrophenyl azide (36.1 mg, 0.220 mmol). Reaction time was 16 h. The product was purified by flash column chromatography (first DCM, followed by EtOAc/heptane = 3:2) to afford **8e** (62 mg, 66%) as an off-white semisolid: ^1^H-NMR (400 MHz, CDCl_3_) δ 7.51 (d, *J* = 2.7 Hz, 1H), 7.13 (d, *J* = 8.3 Hz, 1H), 6.75 (dd, *J* = 8.3, 2.7 Hz, 1H), 5.37 (s, 1H), 4.62 (d, *J* = 8.0 Hz, 1H), 4.60–4.54 (m, 1H), 4.53–4.45 (m, 1H), 4.27–4.19 (m, 1H), 3.95–3.88 (m, 1H), 3.86 (s, 3H), 3.04–2.88 (m, 4H), 1.86–1.80 (m, 1H), 1.71–1.62 (m, 3H), 1.61–1.49 (m, 3H), 1.47 (s, 3H), 1.43–1.35 (m, 1H), 1.34–1.27 (m, 2H), 1.22–1.15 (m, 1H), 1.06–0.95 (m, 1H), 0.90–0.86 (m, 6H). ^13^C-NMR (101 MHz, CDCl_3_) δ 159.10, 143.39, 134.39, 129.97, 129.15, 125.68, 113.95, 107.26, 106.62, 101.41, 97.23, 82.71, 68.49, 55.62, 48.52, 45.22, 44.10, 39.95, 35.25, 34.54, 34.50, 32.79, 27.96, 23.85, 22.15, 19.63, 19.32, 18.74.

*5-Phenyl-1-(2-(((3R,6R,8aS,9R,10R,12R,12aR)-3,6,9-trimethyldecahydro-12H-3,12-epoxy[1,2]dioxepino[4,3-i]isochromen-10-yl)oxy)ethyl)-1H-1,2,3-triazole* (**8f**): **5** (60 mg, 0.183 mmol), acetophenone (22 mg, 0.183 mmol), 4-nitrophenyl azide (36.1 mg, 0.220 mmol). Reaction time was 24 h. The product was purified by flash column chromatography (at first DCM followed by EtOAc/heptane = 3:2) to give **8f** (41 mg, 49%) as an off-white semisolid: ^1^H-NMR (400 MHz, CDCl_3_) δ 7.69 (s, 1H), 7.53–7.44 (m, 5H), 5.35 (s, 1H), 4.64–4.46 (m, 3H), 4.32–4.21 (m, 1H), 4.12–4.00 (m, 1H), 1.88–1.79 (m, 1H), 1.70–1.62 (m, 3H), 1.61–1.49 (m, 3H), 1.47 (s, 3H), 1.42–1.33 (m, 1H), 1.31–1.22 (m, 2H), 1.21–1.12 (m, 1H), 1.06–0.94 (m, 1H), 0.89–0.81 (m, 6H). ^13^C-NMR (101 MHz, CDCl_3_) δ 138.90, 132.92, 129.45, 129.26, 129.10, 127.25, 107.23, 101.60, 97.23, 82.71, 68.08, 48.04, 45.27, 44.12, 39.70, 35.27, 34.57, 34.53, 32.79, 23.86, 22.17, 19.19, 18.76.

*5-(3,4-Dibromophenyl)-1-(2-(((3R,6R,8aS,9R,10R,12R,12aR)-3,6,9-trimethyldecahydro-12H-3,12-epoxy[1,2]dioxepino[4,3-i]isochromen-10-yl)oxy)ethyl)-1H-1,2,3-triazole* (**8g**): 5 (60 mg, 0.183 mmol), 1-(3,4-dibromophenyl)ethan-1-one (51 mg, 0.183 mmol), 4-nitrophenyl azide (36.1 mg, 0.220 mmol). Reaction time was 36 h. Flash column chromatography purification (DCM first, followed by EtOAc/heptane = 3:2) afforded **8g** (42 mg, 37%) as an off-white solid: m.p. 105–106 °C; ^1^H-NMR (400 MHz, CDCl_3_) δ 7.83 (d, *J* = 2.0 Hz, 1H), 7.73–7.69 (m, 2H), 7.32 (dd, *J* = 8.3, 2.1 Hz, 1H), 5.31 (s, 1H), 4.60–4.52 (m, 3H), 4.28–4.22 (m, 1H), 4.07–4.00 (m, 1H), 1.86–1.80 (m, 1H), 1.71–1.64 (m, 2H), 1.62–1.56 (m, 3H), 1.54–1.49 (m, 1H), 1.46 (s, 3H), 1.32–1.22 (m, 3H), 1.19–1.10 (m, 2H), 1.04–0.95 (m, 1H), 0.88 (d, *J* = 5.7 Hz, 3H), 0.84 (d, *J* = 7.1 Hz, 3H). ^13^C-NMR (151 MHz, CDCl_3_) δ 134.32, 134.22, 133.20, 129.19, 128.04, 126.35, 125.68, 107.26, 101.58, 97.21, 82.71, 68.42, 48.52, 45.24, 44.09, 39.71, 35.27, 34.56, 34.53, 32.81, 23.87, 22.17, 19.22, 18.78.

*5-(Naphthalen-1-yl)-1-(2-(((3R,6R,8aS,9R,10R,12R,12aR)-3,6,9-trimethyldecahydro-12H-3,12-epoxy[1,2]dioxepino[4,3-i]isochromen-10-yl)oxy)ethyl)-1H-1,2,3-triazole* (**8h**): 5 (60 mg, 0.183 mmol), 1-(naphthalen-1-yl)ethan-1-one (31.2 mg, 0.183 mmol), 4-nitrophenyl azide (36.1 mg, 0.220 mmol). Reaction time was 36 h. The product was purified by flash column chromatography (first DCM, then EtOAc/heptane = 3:2) to afford **8h** (49 mg, 53%) as an off-white semisolid: ^1^H-NMR (400 MHz, CDCl_3_) δ 7.97 (d, *J* = 8.2 Hz, 1H), 7.92 (d, *J* = 7.7 Hz, 1H), 7.79 (s, 1H), 7.58–7.46 (m, 5H), 5.24 (s, 1H), 4.51 (d, *J* = 8.0 Hz, 1H), 4.42–4.34 (m, 2H), 4.13–4.04 (m, 1H), 3.92–3.83 (m, 1H), 1.84–1.78 (m, 1H), 1.72–1.58 (m, 3H), 1.57–1.45 (m, 3H), 1.41 (s, 3H), 1.31–1.23 (m, 1H), 1.19–1.09 (m, 3H), 1.01–0.93 (m, 1H), 0.89–0.80 (m, 6H). ^13^C-NMR (101 MHz, CDCl_3_) δ 136.53, 134.52, 133.76, 132.25, 130.29, 129.05, 128.66, 127.34, 126.66, 125.27, 125.00, 124.59, 107.13, 101.35, 97.16, 82.65, 67.70, 48.24, 45.26, 44.05, 39.52, 35.23, 34.56, 34.52, 32.70, 23.83, 22.15, 19.25, 18.76.

*2-Methoxy-4-(1-(2-(((3R,6R,8aS,9R,10R,12R,12aR)-3,6,9-trimethyldecahydro-12H-3,12-epoxy[1,2]dioxepino[4,3-i]isochromen-10-yl)oxy)ethyl)-1H-1,2,3-triazol-5-yl)phenol* (**8i**): 5 (60 mg, 0.183 mmol), 1-(4-hydroxy-3-methoxyphenyl)ethan-1-one (30.5 mg, 0.183 mmol), 4-nitrophenyl azide (36.1 mg, 0.220 mmol). Reaction time was 36 h. The product was purified by flash column chromatography (first DCM, followed by EtOAc/heptane = 3:2) to give **8i** (35 mg, 38%) as an off-white semisolid: ^1^H-NMR (400 MHz, CDCl_3_) δ 7.64 (s, 1H), 7.01 (s, 3H), 5.86 (s, 1H), 5.34 (s, 1H), 4.59 (d, *J* = 7.9 Hz, 1H), 4.57–4.44 (m, 2H), 4.31–4.21 (m, 1H), 4.11–4.02 (m, 1H), 3.93 (s, 3H), 1.88–1.80 (m, 1H), 1.70–1.58 (m, 3H), 1.57–1.50 (m, 3H), 1.47 (s, 3H), 1.40–1.31 (m, 1H), 1.30–1.20 (m, 2H), 1.18–1.10 (m, 1H), 1.06–0.96 (m, 1H), 0.89–0.81 (m, 6H). ^13^C-NMR (101 MHz, CDCl_3_) δ 146.91, 138.98, 132.62, 122.83, 118.92, 115.07, 111.85, 107.27, 101.57, 97.25, 82.69, 68.20, 56.29, 47.85, 45.25, 44.10, 39.83, 35.27, 34.56, 34.53, 32.81, 29.84, 23.84, 22.17, 19.19, 18.77.

*5-Benzyl-1-(2-(((3R,6R,8aS,9R,10R,12R,12aR)-3,6,9-trimethyldecahydro-12H-3,12-epoxy[1,2]dioxepino[4,3-i]isochromen-10-yl)oxy)ethyl)-4,5,6,7-tetrahydro-1H-[1,2,3]triazolo[4,5-c]pyridine* (**8j**): **5** (60 mg, 0.183 mmol), 1-benzylpiperidin-4-one (34.7 mg, 0.183 mmol), 4-nitrophenyl azide (36.1 mg, 0.220 mmol). Reaction time was 16 h. The product was purified by flash column chromatography (first DCM, next EtOAc/heptane = 3:2) to afford **8j** (61 mg, 63%) as an off-white semisolid: ^1^H-NMR (400 MHz, CDCl_3_) δ 7.38–7.28 (m, 5H), 5.37 (s, 1H), 4.60 (d, *J* = 7.9 Hz, 1H), 4.52–4,46 (m, 1H), 4.42–4.34 (m, 1H), 4.23–4.17 (m, 1H), 3.90–3.82 (m, 1H), 3.75 (s, 2H), 3.72–3.64 (m, 2H), 2.85–2.68 (m, 4H), 1.85–1.81 (m, 1H), 1.70–1.53 (m, 6H), 1.47 (s, 3H), 1.39–1.36 (m, 1H), 1.34–1.26 (m, 1H), 1.95–1.12 (m, 2H), 1.08–0.99 (m, 1H), 0.92–0.83 (m, 6H). ^13^C-NMR (101 MHz, CDCl_3_) δ 142.03, 138.18, 131.76, 129.08, 128.52, 127.46, 107.26, 101.43, 97.23, 82.71, 68.54, 61.64, 49.92, 49.39, 48.23, 45.25, 44.14, 39.93, 35.26, 34.55, 34.52, 32.81, 29.82, 23.86, 22.16, 20.99, 19.28, 18.76.

*(1S,3aS,3bR,5aS,10aS,10bS,12aS)-10a,12a-Dimethyl-7-(2-(((3R,6R,8aS,9R,10R,12R,12aR)-3,6,9-trimethyl-decahydro-12H-3,12-epoxy[1,2]dioxepino[4,3-i]isochromen-10-yl)oxy)ethyl)-1,2,3,3a,3b,4,5,5a,6,7,10,10a, 10b,11,12,12a-hexadecahydrocyclopenta[7,8]phenanthro[2,3-d][1,2,3]triazol-1-ol* (**8k**): **5** (60 mg, 0.183 mmol), (5*S*,8*R*,9*S*,10*S*,13*S*,14*S*,17*S*)-17-hydroxy-10,13-dimethylhexadecahydro-3*H*-cyclopenta[*a*]phenanthren-3-one (53.2 mg, 0.183 mmol), 4-nitrophenyl azide (36.1 mg, 0.220 mmol). Reaction time was 16 h. Flash column chromatography (first DCM, followed by EtOAc/heptane = 3:2) yielded **8k** (72 mg, 63%) as an off-white solid: m.p. 96–97 °C; ^1^H-NMR (400 MHz, CDCl_3_) δ 5.39 (s, 1H), 4.61 (d, *J* = 7.9 Hz, 1H), 4.48 (dt, *J* = 14.2, 4.2 Hz, 1H), 4.40–4.31 (m, 1H), 4.20 (dt, *J* = 8.9, 4.4 Hz, 1H), 3.91–3.85 (m, 1H), 3.66 (t, *J* = 8.5 Hz, 1H), 2.85 (d, *J* = 15.3 Hz, 1H), 2.55 (dd, *J* = 16.2, 5.0 Hz, 1H), 2.30 (t, *J* = 13.6 Hz, 2H), 2.11–2.03 (m, 1H), 1.89 - 1.82 (m, 2H), 1.75–1.51 (m, 10H), 1.48–1.47 (m, 3H), 1.45–1.38 (m, 3H), 1.32–1.25 (m, 3H), 1.20–1.12 (m, 3H), 1.05–0.96 (m, 2H), 0.91–0.87 (m, 6H), 0.76 (s, 3H), 0.72 (s, 3H). ^13^C-NMR (151 MHz, CDCl_3_) δ 142.96, 131.85, 107.28, 101.25, 97.27, 82.74, 81.99, 68.40, 53.86, 50.95, 48.03, 45.26, 44.14, 43.00, 42.39, 39.98, 36.96, 36.78, 36.31, 35.75, 35.32, 34.56, 34.54, 32.84, 31.34, 30.60, 29.05, 25.01, 23.87, 23.58, 22.18, 20.93, 19.39, 18.78, 11.73, 11.17.

*(1R,3aS,5aR,5bR,7aR,12aR,12bR,14aR,14bR)-5a,5b,8,8,12a-Pentamethyl-1-(prop-1-en-2-yl)-9-(2-(((3R,6R,8aS,9R,10R,12R,12aR)-3,6,9-trimethyldecahydro-12H-3,12-epoxy[1,2]dioxepino[4,3-i]isochromen-10-yl)-oxy)ethyl)-2,3,4,5,5a,5b,6,7,7a,8,9,12,12a,12b,13,14,14a,14b-octadecahydrocyclopenta[7,8]chryseno[2,3-d][1,2,3]triazole-3a(1H)-carboxylic acid* (**8l**): 5 (60 mg, 0.183 mmol), (1*R*,3a*S*,5a*R*,5b*R*,7a*R*,11a*R*, 11b*R*,13a*R*,13b*R*)-5a,5b,8,8,11a*-*pentamethyl-9-oxo-1-(prop-1-en-2-yl)icosahydro-3a*H*-cyclopenta[*a*]chrysene-3a-carboxylic acid (83 mg, 0.183 mmol), 4-nitrophenyl azide (36.1 mg, 0.220 mmol). Reaction time was 16 h. The product was purified by flash column chromatography (first DCM, followed by EtOAc/heptane = 3:2) to afford **8l** (68 mg, 47%) as an off-white semisolid: ^1^H-NMR (400 MHz, CDCl_3_) δ 5.39 (s, 1H), 4.76 (s, 1H), 4.69–4.63 (m, 2H), 4.59–4.51 (m, 1H), 4.28–4.23 (m, 1H), 4.19–4.11 (m, 1H), 3.06–3.00 (m, 1H), 2.91 (d, *J* = 15.3 Hz, 1H), 2.31–2.23 (m, 2H), 2.12 (d, *J* = 15.3 Hz, 1H), 2.03–1.96 (m, 2H), 1.86–1.76 (m, 3H), 1.71 (s, 3H), 1.69–1.63 (m, 3H), 1.60–1.50 (m, 8H), 1.48 (s, 5H), 1.45–1.39 (m, 2H), 1.37 (d, *J* = 2.5 Hz, 1H), 1.32 (d, *J* = 7.6 Hz, 4H), 1.29–1.22 (m, 9H), 1.18–1.10 (m, 3H), 1.00 (d, *J* = 4.6 Hz, 6H), 0.90 - 0.87 (m, 6H), 0.78 (s, 3H). ^13^C-NMR (101 MHz, CDCl_3_) δ 180.60, 150.37, 141.10, 138.35, 110.01, 107.26, 101.71, 97.31, 82.75, 68.55, 56.48, 54.86, 49.47, 49.31, 49.21, 47.00, 45.30, 44.10, 42.61, 40.75, 39.80, 39.09, 38.59, 38.42, 37.18, 35.29, 34.60, 34.56, 33.76, 33.54, 32.82, 32.22, 30.71, 29.94, 29.84, 28.82, 25.65, 23.90, 22.19, 21.57, 19.55, 19.33, 19.09, 18.80, 16.17, 15.80, 14.83.

*(3R,6R,8aS,9R,12R,12aR)-3,6,9-Trimethyldecahydro-12H-3,12-epoxy[1,2]dioxepino[4,3-i]isochromen-10-yl 4-oxocyclohexane-1-carboxylate* (**9**): dihydroartemisinin (1 gm, 3.52 mmol), dicyclohexyl-methanediimine (0.726 gm, 3.52 mmol), 4-oxocyclohexane-1-carboxylic acid (0.600 gm, 4.22 mmol), *N*,*N*-dimethylpyridin-4-amine (0.064 gm,0.528 mmol.). Reaction time was 18 h. The product was purified by flash column chromatography (DCM/MeOH = 99/1) to give **9** (1.2 gm, 84%) as an off-white semisolid: ^1^H-NMR (400 MHz, CDCl_3_) δ 5.82 (d, *J* = 9.8 Hz, 1H), 5.45 (s, 1H), 2.91–2.75 (m, 1H), 2.60 (ddd, *J* = 9.9, 7.2, 4.6 Hz, 1H), 2.62–2.58 (m, 1H), 2.55–2.45 (m, 1H), 2.39–2.34 (m, 2H), 2.33–2.27 (m, 1H), 2.25–2.20 (m, 1H), 2.14–2.00 (m, 3H), 1.95–1.87 (m, 1H), 1.81–1.75 (m, 2H), 1.67–1.56 (m, 2H), 1.52–1.46 (m, 1H), 1.45–1.41 (m, 3H), 1.39–1.25 (m, 3H), 1.11–1.00 (m, 1H), 0.97 (d, *J* = 6.0 Hz, 3H), 0.86 (d, *J* = 7.1 Hz, 3H). ^13^C-NMR (101 MHz, CDCl_3_) δ 210.13, 173.04, 104.63, 92.27, 91.67, 80.25, 77.48, 76.84, 51.70, 45.39, 40.70, 39.87, 39.66, 37.41, 36.33, 34.21, 31.94, 28.78, 28.05, 26.06, 24.71, 22.13, 20.34, 12.30.

*(3R,6R,8aS,9R,10S,12R,12aR)-3,6,9-Trimethyldecahydro-12H-3,12-epoxy[1,2]dioxepino[4,3-i]isochromen-10-yl 1-(4-methylbenzyl)-4,5,6,7-tetrahydro-1H-benzo[d][1,2,3]triazole-5-carboxylate* (**11a**): **9** (60 mg, 0.147 mmol), *p*-tolylmethanamine (18 mg, 0.147 mmol), 4-nitrophenyl azide (28.9 mg, 0.176 mmol). Reaction time was 16 h. Flash column chromatography (first DCM, followed by EtOAc/heptane = 3:2) afforded purified **11a** (53 mg, 67%) as an off-white semisolid: ^1^H-NMR (400 MHz, CDCl_3_) δ 7.14 (d, *J* = 7.8 Hz, 2H), 7.08 (d, *J* = 7.8 Hz, 2H), 5.83–5.69 (m, 1H), 5.48–5.29 (m, 3H), 3.20–3.05 (m, 1H), 3.03–2.91 (m, 1H), 2.86–2.76 (m, 1H), 2.68–2.50 (m, 2H), 2.44–2.36 (m, H), 2.33 (s, 3H), 2.29–2.12 (m, 1H), 2.07–1.99 (m, 1H), 1.95–1.85 (m, 2H), 1.79–1.69 (m, 2H), 1.66–1.57 (m, 2H), 1.54–1.45 (m, 1H), 1.42 (d, *J* = 1.6 Hz, 3H), 1.38–1.25 (m, 3H), 0.96 (d, *J* = 5.8 Hz, 3H), 0.80 (d, *J* = 7.1 Hz, 3H). ^13^C-NMR (151 MHz, CDCl_3_) δ 173.24, 172.94, 141.10, 141.06, 136.27, 136.21, 132.13, 127.09, 127.02, 122.90, 122.73, 122.40, 122.24, 119.72, 119.63, 118.14, 118.05, 111.69, 111.62, 111.23, 104.76, 104.72, 92.59, 92.53, 91.82, 80.38, 80.22, 51.74, 51.70, 48.99, 48.80, 45.35, 39.53, 39.38, 37.40, 36.38, 34.18, 32.02, 31.90, 26.77, 26.68, 26.03, 25.92, 25.02, 24.80, 24.69, 24.35, 23.84, 22.15, 20.34, 18.23, 18.04, 12.32, 12.22.

*(3R,6R,8aS,9R,10S,12R,12aR)-3,6,9-Trimethyldecahydro-12H-3,12-epoxy[1,2]dioxepino[4,3-i]isochromen-10-yl 1-(3,4,5-trimethoxybenzyl)-4,5,6,7-tetrahydro-1H-benzo[d][1,2,3]triazole-5-carboxylate* (**11b**): **9** (60 mg, 0.147 mmol), (3,4,5-trimethoxyphenyl)methanamine (29 mg, 0.147 mmol), 4-nitrophenyl azide (28.9 mg, 0.176 mmol). Reaction time was 16 h. The product was purified by flash column chromatography (DCM at first, followed by EtOAc/heptane = 3:2) to afford **11b** (55 mg, 61%) as an off-white solid: mp 80–81 °C; ^1^H-NMR (400 MHz, CDCl_3_) δ 6.40 (d, *J* = 6.8 Hz, 2H), 5.82–5.74 (m, 1H), 5.43 (t, *J* = 4.6 Hz, 1H), 5.39–5.28 (m, 2H), 3.83 (d, *J* = 1.0 Hz, 3H), 3.81 (d, *J* = 1.5 Hz, 6H), 3.20–2.94 (m, 2H), 2.88–2.81 (m, 1H), 2.74–2.54 (m, 2H), 2.52–2.42 (m, 1H), 2.42–2.33 (m, 1H), 2.31–2.20 (m, 1H), 2.02–1.86 (m, 2H), 1.79–1.68 (m, 2H), 1.66–1.59 (m, 1H), 1.42 (d, *J* = 3.5 Hz, 3H), 1.32–1.23 (m, 4H), 0.97 (d, *J* = 5.8 Hz, 3H), 0.90–0.84 (m, 2H), 0.81 (d, *J* = 7.1 Hz, 3H). ^13^C-NMR (101 MHz, CDCl_3_) δ 173.06, 172.85, 153.77, 142.64, 142.33, 138.12, 131.47, 131.12, 130.33, 130.30, 104.73, 104.66, 104.61, 104.57, 92.30, 91.65, 80.22, 80.20, 60.97, 56.34, 52.31, 52.28, 51.67, 45.37, 45.33, 39.82, 39.47, 37.39, 36.31, 34.18, 31.92, 31.87, 29.81, 26.03, 25.47, 24.99, 24.68, 24.20, 22.09, 20.31, 19.29, 18.87, 12.24, 12.16.

*(3R,6R,8aS,9R,10S,12R,12aR)-3,6,9-Trimethyldecahydro-12H-3,12-epoxy[1,2]dioxepino[4,3-i]isochromen-10-yl 1-(benzo[d][1,3]dioxol-5-ylmethyl)-4,5,6,7-tetrahydro-1H-benzo[d][1,2,3]triazole-5-carboxylate* (**11c**): **9** (60 mg, 0.147 mmol), benzo[*d*][1,3]dioxol-5-ylmethanamine (22.2 mg, 0.147 mmol), 4-nitrophenyl azide (28.9 mg, 0.176 mmol). Reaction time was 16 h. The product was purified by flash column chromatography (first DCM, followed by EtOAc/heptane = 3:2) to give **11c** (51 mg, 61%) as an off-white semisolid: ^1^H-NMR (400 MHz, CDCl_3_) δ 6.76 (dd, *J* = 6.9, 1.7 Hz, 1H), 6.68 (dt, *J* = 3.7, 1.6 Hz, 2H), 5.96 (s, 2H), 5.83–5.73 (m, 1H), 5.43 (d, *J* = 3.0 Hz, 1H), 5.39–5.25 (m, 2H), 3.18–3.06 (m, 1H), 3.04–2.92 (m, 1H), 2.87–2.78 (m, 1H), 2.69–2.54 (m, 2H), 2.47–2.33 (m, 2H), 2.28–2.19 (m, 1H), 2.06–1.86 (m, 3H), 1.79–1.69 (m, 2H), 1.66–1.58 (m, 1H), 1.51–1.45 (m, 1H), 1.42 (d, *J* = 1.9 Hz, 3H), 1.34–1.24 (m, 2H), 1.07–0.98 (m, 1H), 0.96 (d, *J* = 5.9 Hz, 3H), 0.91–0.84 (m, 1H), 0.83–0.78 (m, 3H). ^13^C-NMR (101 MHz, CDCl_3_) δ 173.09, 172.92, 148.40, 147.87, 142.33, 131.34, 128.43, 121.33, 108.54, 108.21, 108.19, 104.59, 104.55, 101.45, 92.29, 91.64, 80.22, 80.19, 51.98, 51.67, 45.38, 45.33, 39.47, 37.38, 37.36, 36.31, 34.18, 31.92, 31.86, 26.04, 25.50, 24.99, 24.67, 24.23, 22.08, 20.31, 19.36, 18.86, 12.15.

*(3R,6R,8aS,9R,10S,12R,12aR)-3,6,9-Trimethyldecahydro-12H-3,12-epoxy[1,2]dioxepino[4,3-i]isochromen-10-yl 1-(2-(1*H*-indol-3-yl)ethyl)-4,5,6,7-tetrahydro-1H-benzo[d][1,2,3]triazole-5-carboxylate* (**11d**): **9** (60 mg, 0.147 mmol), 2-(1*H*-indol-3-yl)ethan-1-amine (25.5 mg, 0.147 mmol), 4-nitrophenyl azide (28.9 mg, 0.176 mmol). Reaction time was 16 h. The product was purified by flash column chromatography (first DCM, followed by EtOAc/heptane = 3:2) to afford **11d** (39 mg, 46%) as an off-white semisolid: ^1^H-NMR (400 MHz, CDCl_3_) δ 8.42 (d, *J* = 7.1 Hz, 1H), 7.43–7.33 (m, 2H), 7.22–7.16 (m, 1H), 7.11–7.06 (m, 1H), 6.70 (dd, *J* = 51.1, 2.3 Hz, 1H), 5.73 (dd, *J* = 9.9, 6.4 Hz, 1H), 5.47 (d, *J* = 13.4 Hz, 1H), 4.53–4.31 (m, 2H), 3.37–3.24 (m, 2H), 3.06–2.87 (m, 2H), 2.70–2.55 (m, 2H), 2.42–2.32 (m, 1H), 2.03 (ddd, *J* = 14.5, 7.3, 4.2 Hz, 1H), 1.93–1.85 (m, 3H), 1.82–1.74 (m, 2H), 1.72–1.61 (m, 4H), 1.54–1.41 (m, 1H), 1.41–1.34 (m, 4H), 1.34–1.25 (m, 2H), 0.97 (d, *J* = 5.6 Hz, 3H), 0.83 (d, *J* = 7.1 Hz, 3H). ^13^C-NMR (151 MHz, CDCl_3_) δ 173.24, 172.94, 141.10, 141.06, 136.27, 136.21, 132.13, 127.09, 127.02, 122.90, 122.73, 122.40, 122.24, 119.72, 119.63, 118.14, 118.05, 111.69, 111.62, 111.23, 104.76, 104.72, 92.59, 92.53, 91.82, 80.38, 80.22, 51.74, 51.70, 48.99, 48.80, 45.35, 39.53, 39.38, 37.40, 36.38, 34.18, 32.02, 31.90, 26.77, 26.68, 26.03, 25.92, 25.02, 24.80, 24.69, 24.35, 23.84, 22.15, 20.34, 18.23, 18.04, 12.32, 12.22.

## 4. Conclusions

In conclusion, a series of newly functionalized artemisinin derivatives has been prepared by using a organocatalytic multicomponent reaction. The starting precursors **5** and **9** were used for triazolization reactions resulting in the formation of fused and 1,5-disubstituted 1,2,3-triazole derivatives. All derivatives were screened against HIV wt and three of the molecules exhibited moderate activity. The β-tetralone derivatives **8b**, **8c**, and **8e** were inhibitory to HIV-1 replication in cell culture with a limited cytotoxicity. However, no inhibitory activity was observed against HIV-2 and an NNRTI-resistant double RT mutant (K103N; Y181C) HIV-1 strain (RES056), pointing at an NNRTI-type mode of action for the active derivatives. Further studies on modification of artemisinin by triazolization reactions are under investigation and will be reported in due time.

## Figures and Tables

**Table 1:**
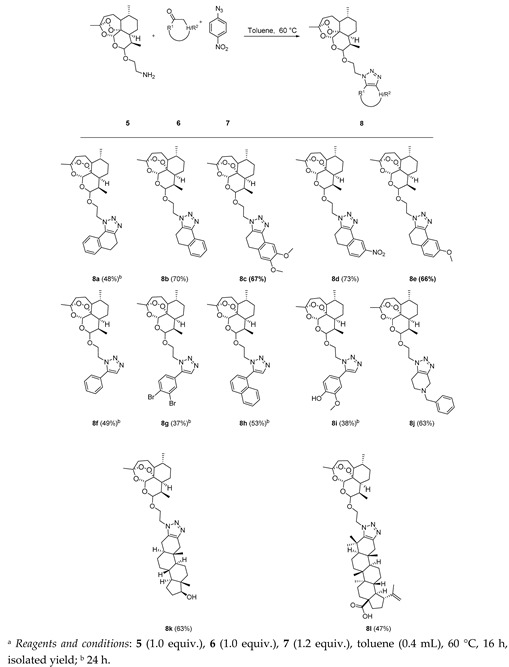
Substrate scope with respect to ketones ^a^.

**Table 2:**
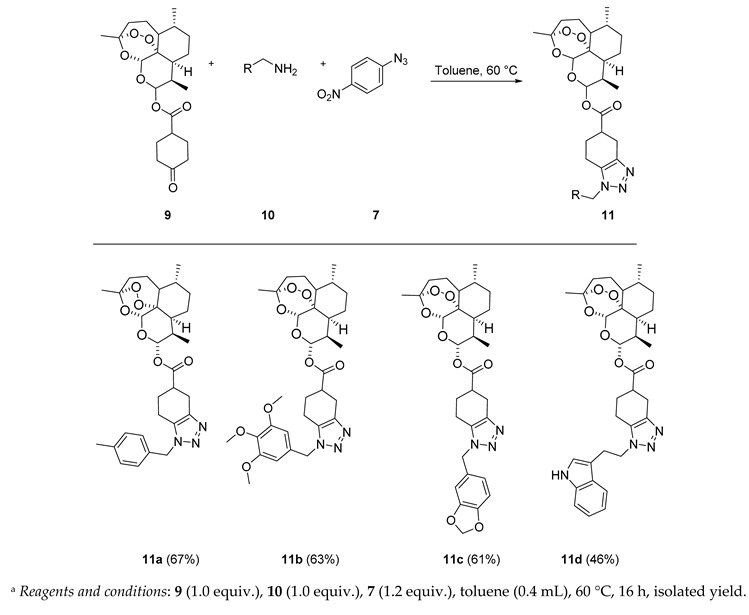
Substrate scope with respect to amines ^a^.

**Table 3:** In vitro anti-HIV activity and cytotoxicity of triazole derivatives of dihydroartemisinin.

Compound	IC_50_ (µM) ^a^			CC_50_ (µM) ^b^
	HIV-1 Strain III_B_	HIV-2 Strain ROD	HIV-1 Strain RES056	
**8a**	>10.3	>10.3	NT ^c^	>10.3
**8b**	2.78	>31.5	>31.5	31.5
**8c**	4.06	>21.2	>21.2	21.2
**8e**	5.18	>14.9	>14.9	14.9
Azidothymidine	0.0064	0.0082	0.0071	>7.50
Lamivudine	2.53	9.90	NT	>87.2
Didanosine	76.0	82.1	NT	>212
Nevirapine	0.075	>15.0	>15.0	>15.0
Efavirenz	0.0024	>6.3	0.18	>6.3
Etravirine	0.0034	>2.29	0.045	2.29

^a^ IC_50_: concentration of compound required to achieve 50% protection of MT-4 cell cultures against HIV-1-induced cytotoxicity, as determined by the MTT (3-(4,5-dimethylthiazol-2-yl)-2,5-diphenyltetrazolium bromide) method; ^b^ CC_50_: concentration required to reduce the viability of mock-infected cell cultures by 50%, as determined by the MTT method; ^c^ Not tested.

## Supplementary Materials

Supplementary materials are available online.
