# Supplementary material for: Application of the Triazolization Reaction to Afford Dihydroartemisinin Derivatives with Anti-HIV Activity
Source: Molecules. 2017 Feb 17;22(2):303. doi: 10.3390/molecules22020303 (PMC6155659; doi:10.3390/molecules22020303)
Supplement: Supplementary file 1 [file molecules-22-00303-s001.pdf]

Supplementary Materials: Application of the Triazolization Reaction to Afford Derivatives of Dihydroartemisinin Having Anti-HIV Activity

Sampad Jana, Shabina Iram, Joice Thomas, Muhammad Qasim Hayat, Christophe Pannecouque and Wim Dehaen

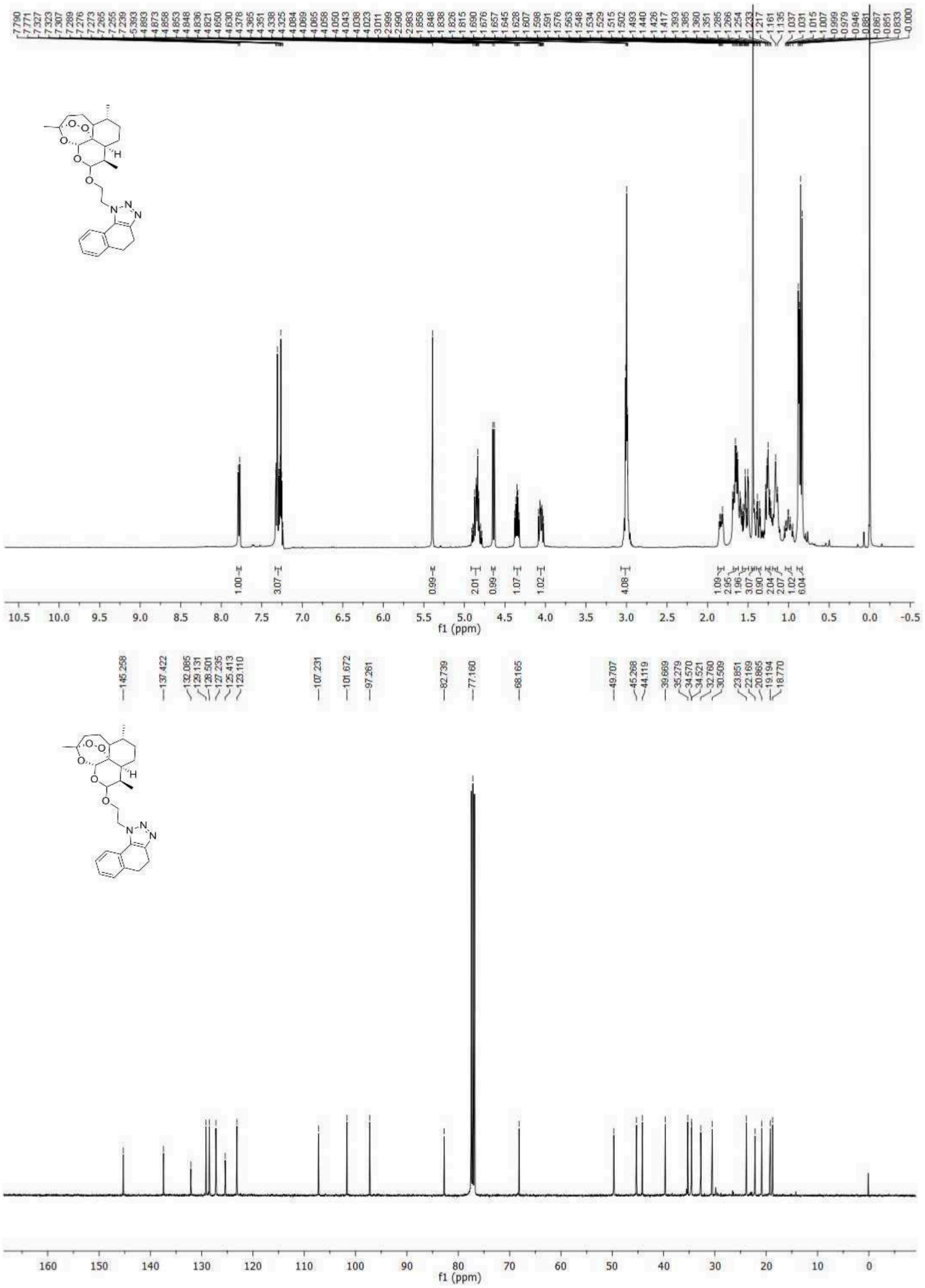

Figure S1. ^{1}H and ^{13}C spectra of **8a**.

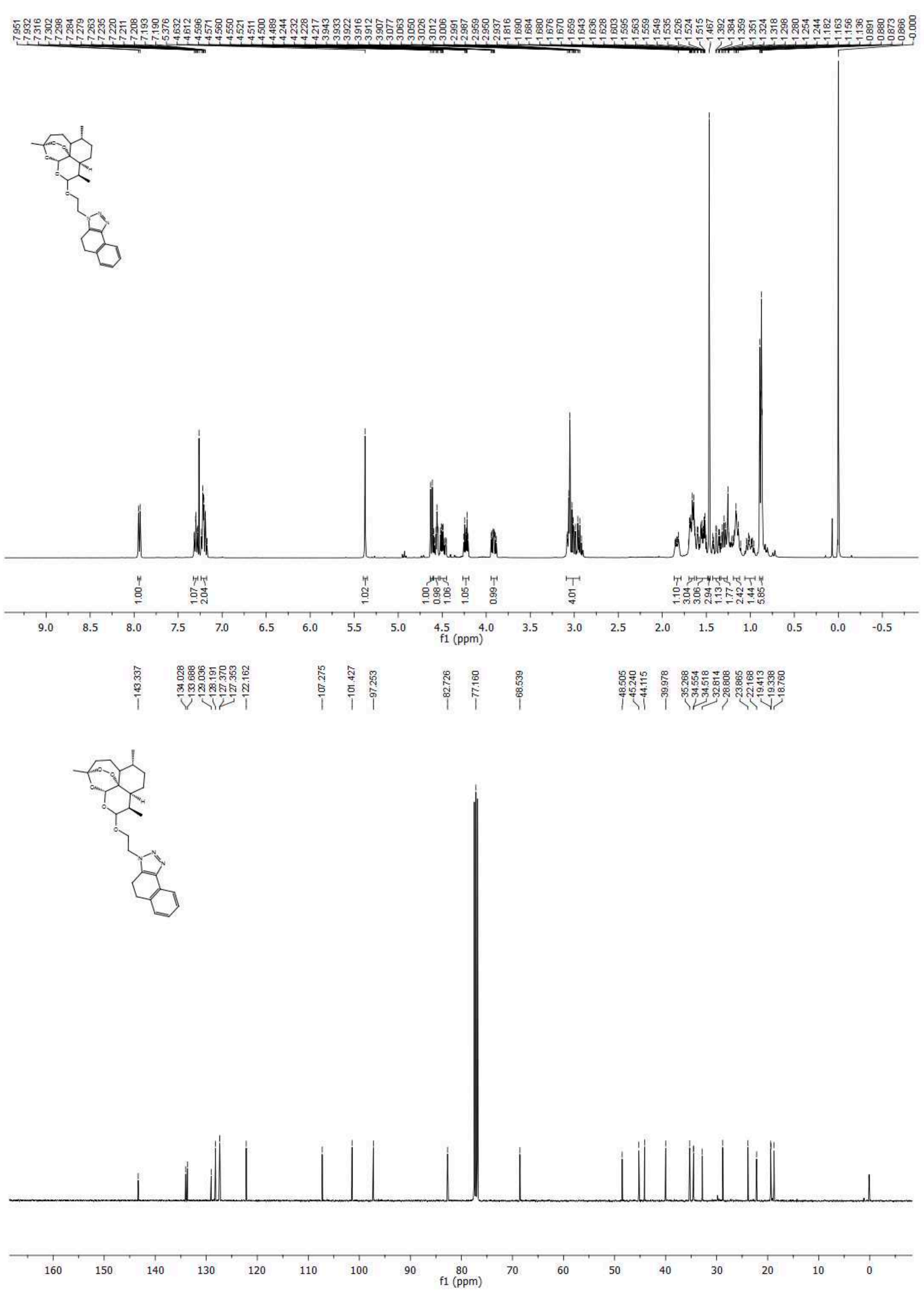

Figure S2. ^{1}H and ^{13}C spectra of **8b**.

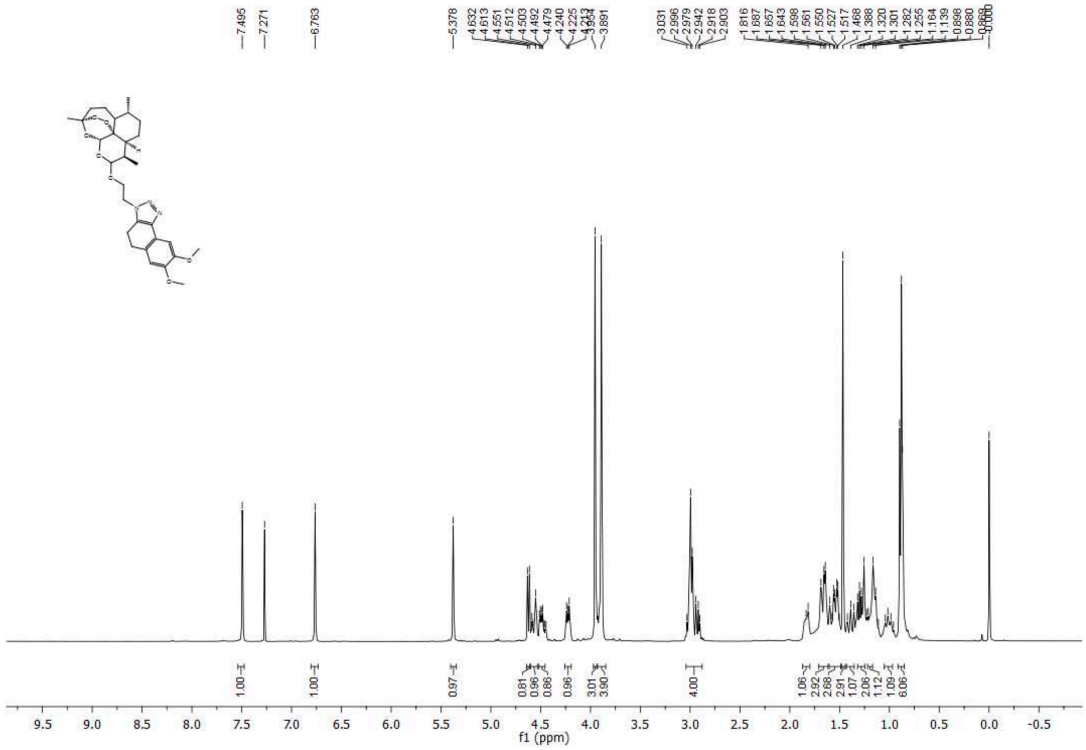

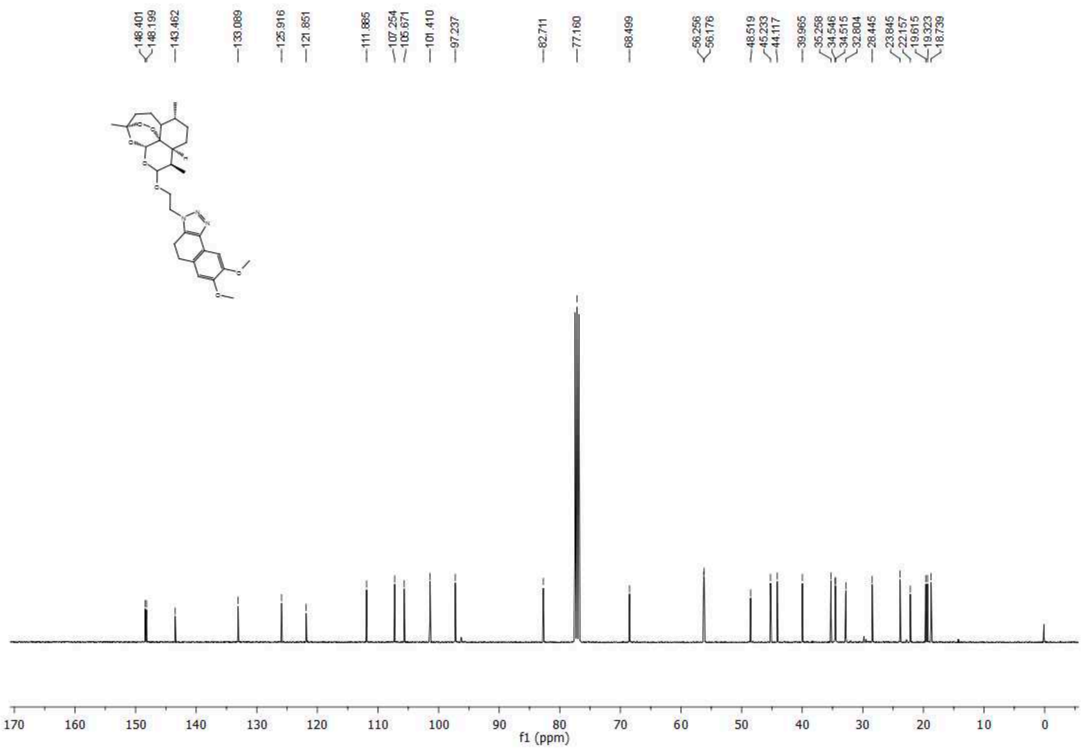

Figure S3. ^{1}H and ^{13}C spectra of **8c**.

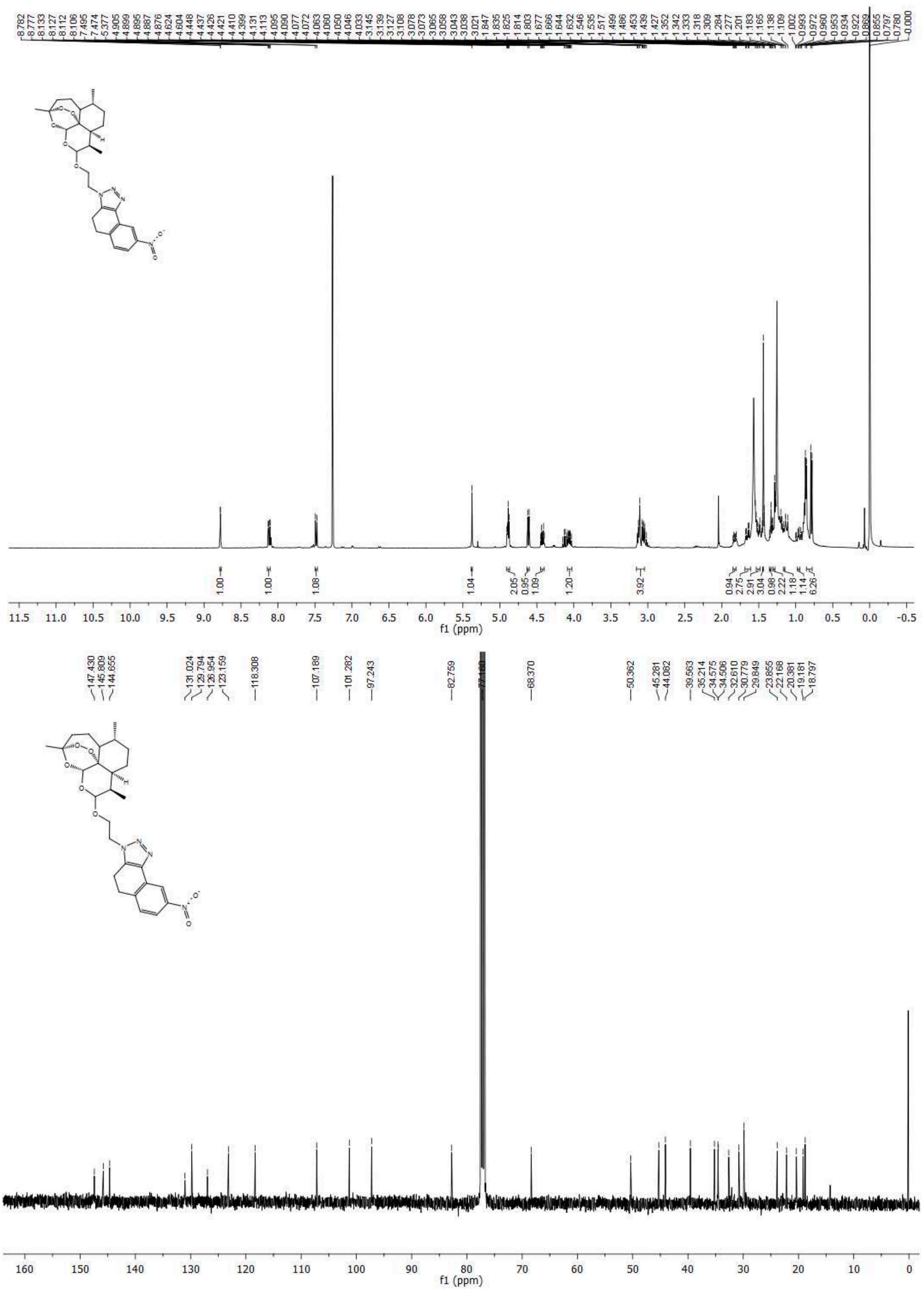

Figure S4. ^{1}H and ^{13}C spectra of **8d**.

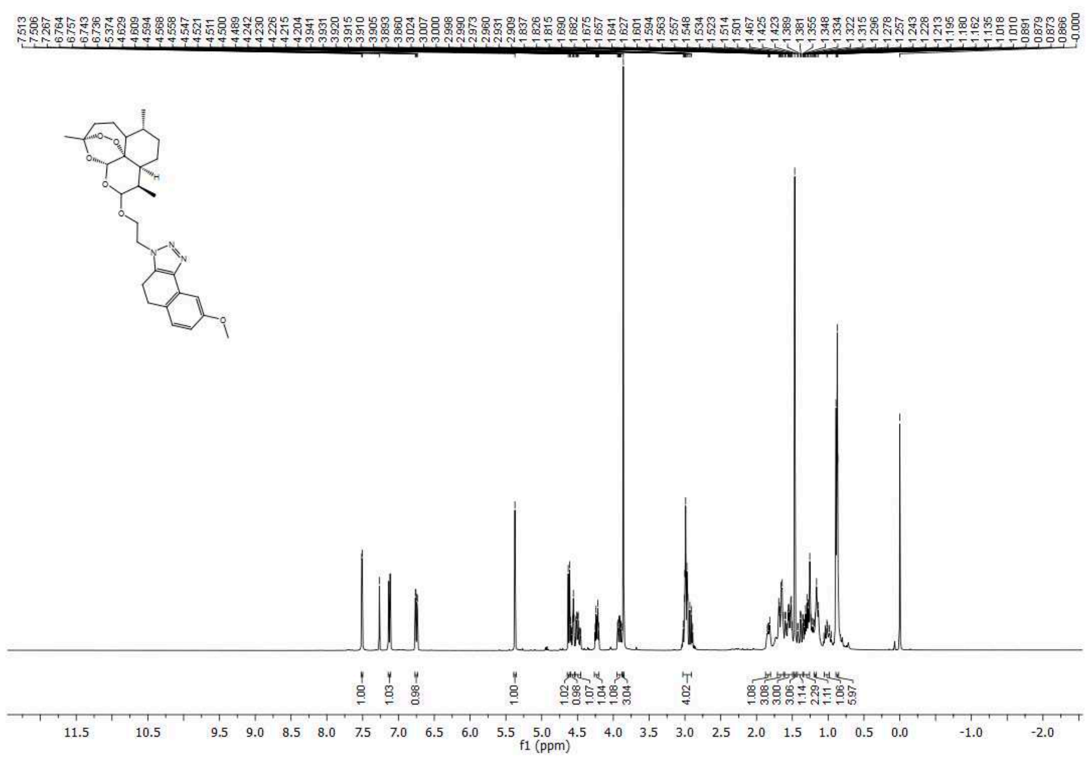

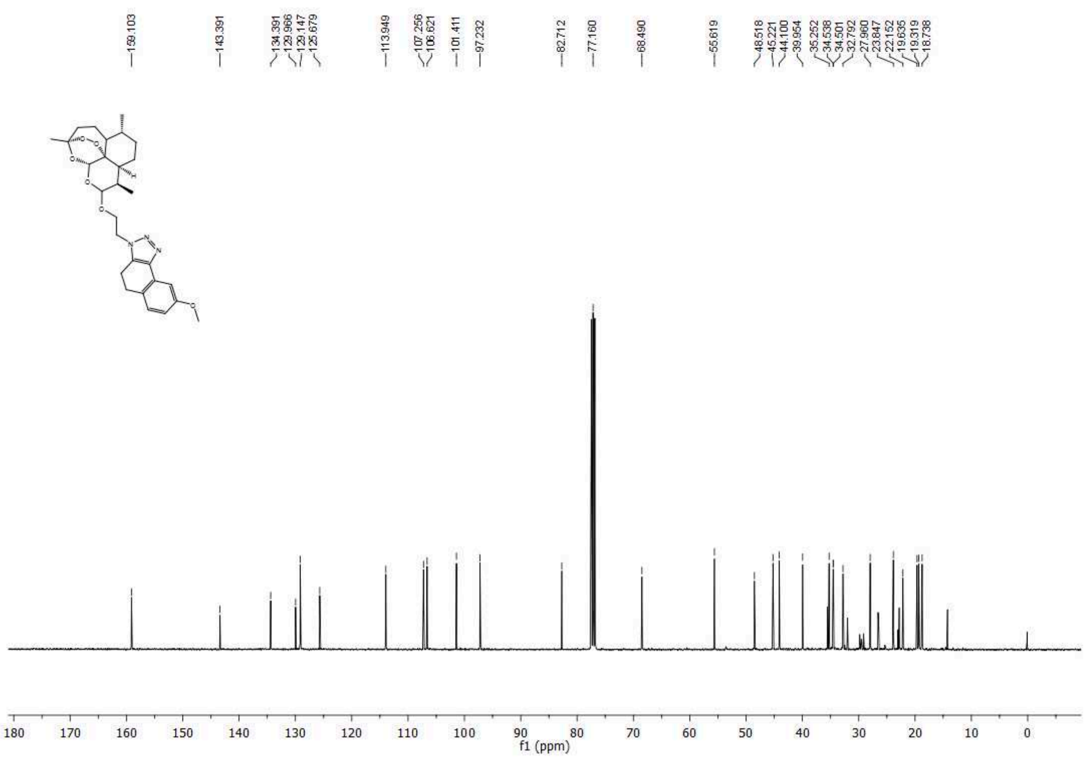

Figure S5. ^{1}H and ^{13}C spectra of **8e**.

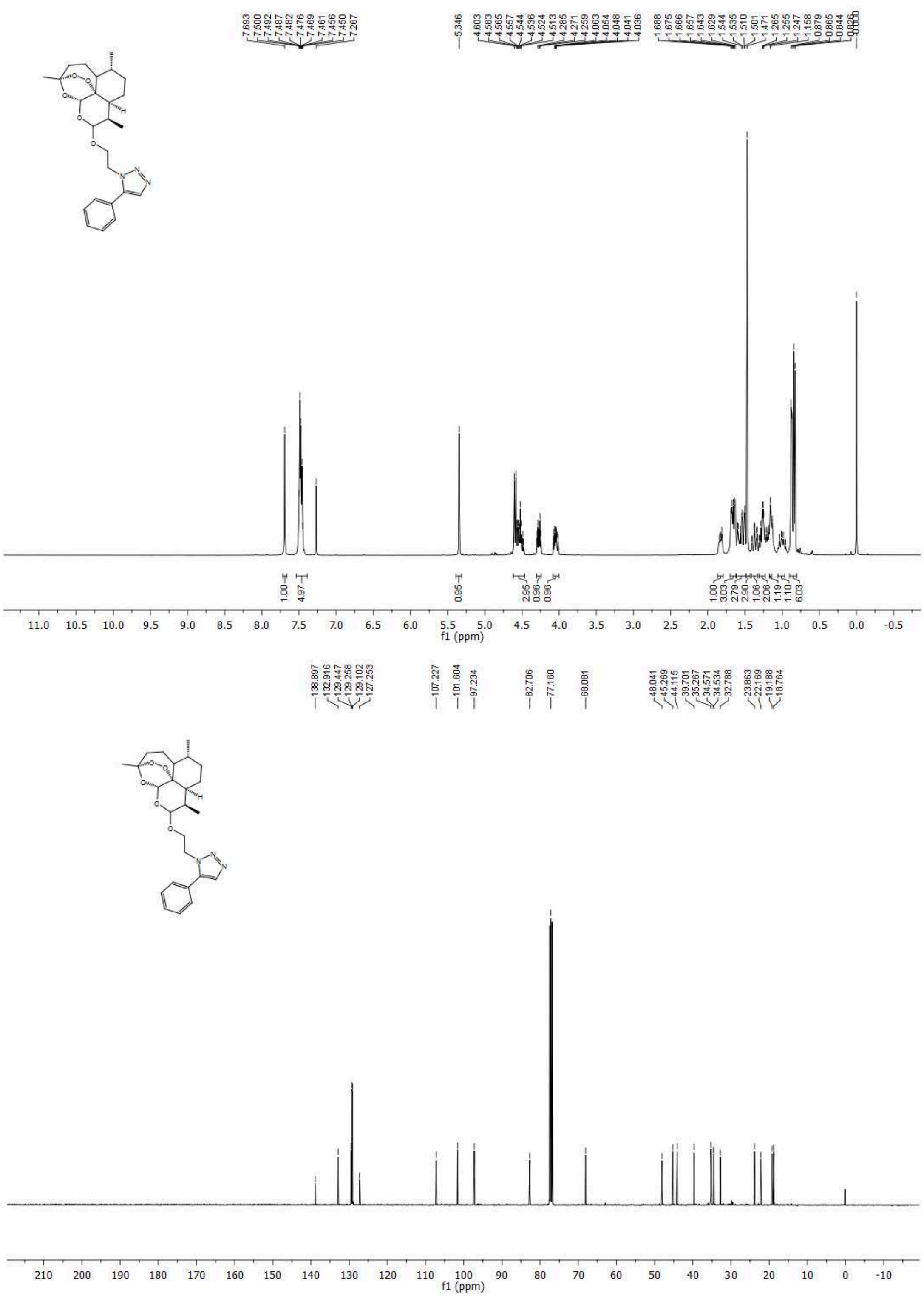

Figure S6. ^{1}H and ^{13}C spectra of **8f**.

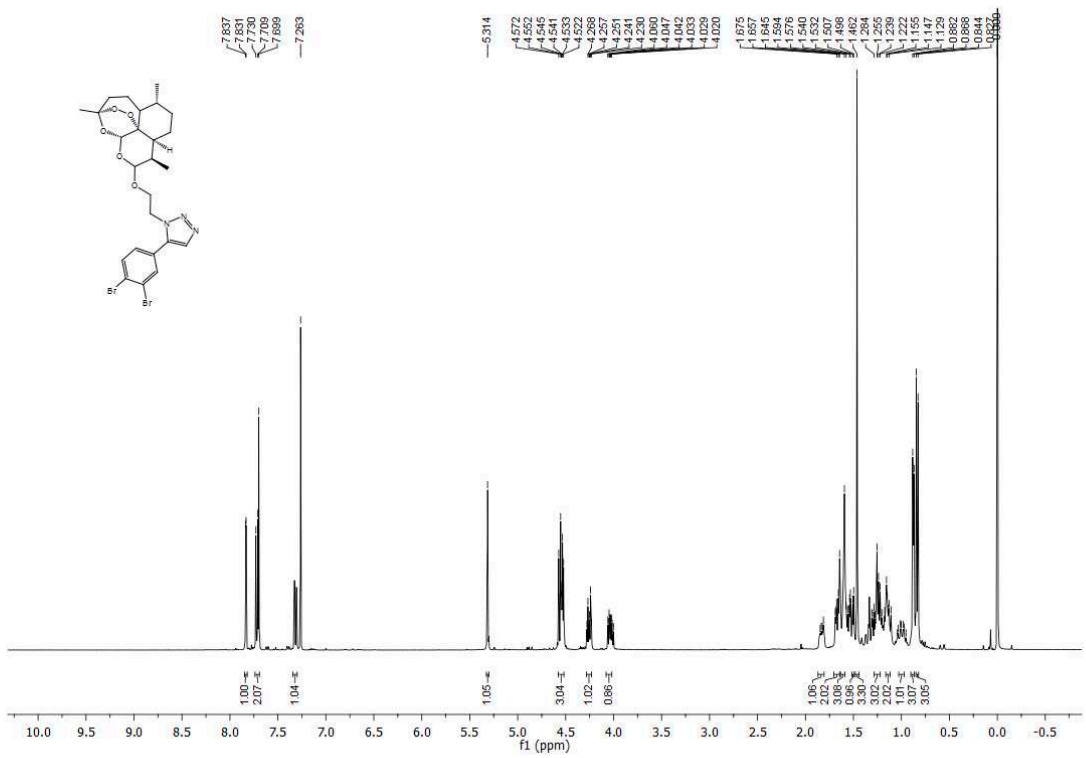

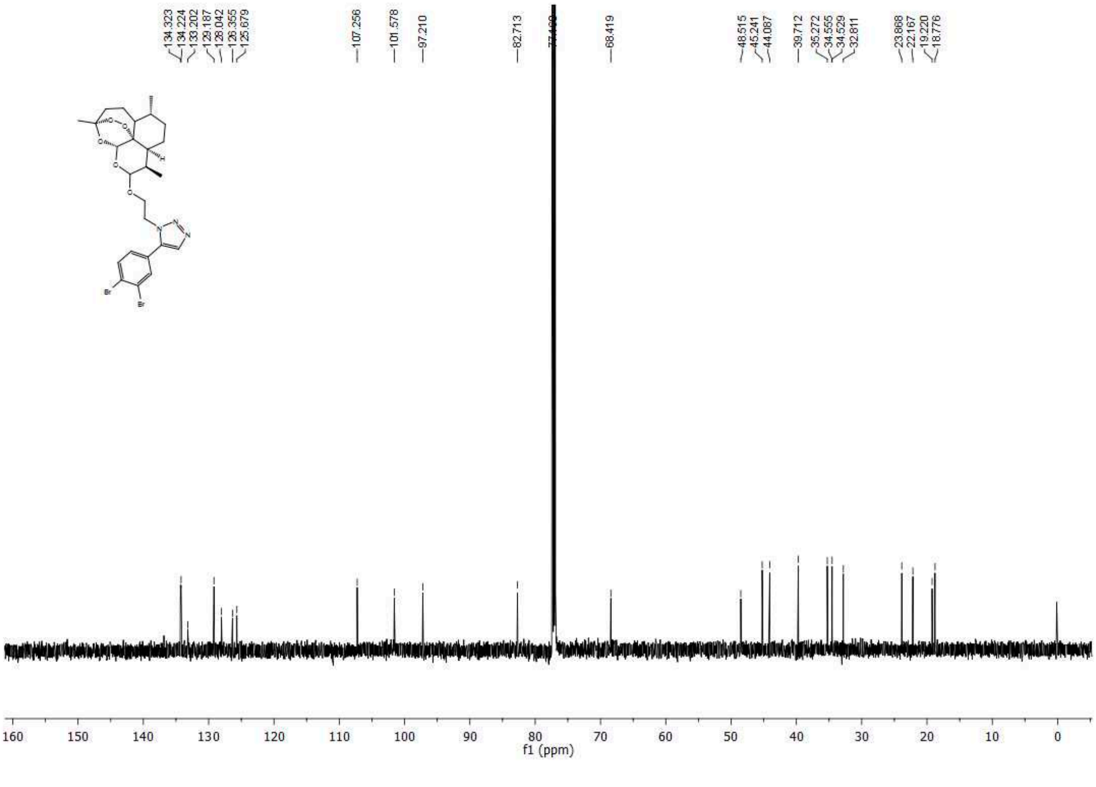

Figure S7. ^{1}H and ^{13}C spectra of **8g**.

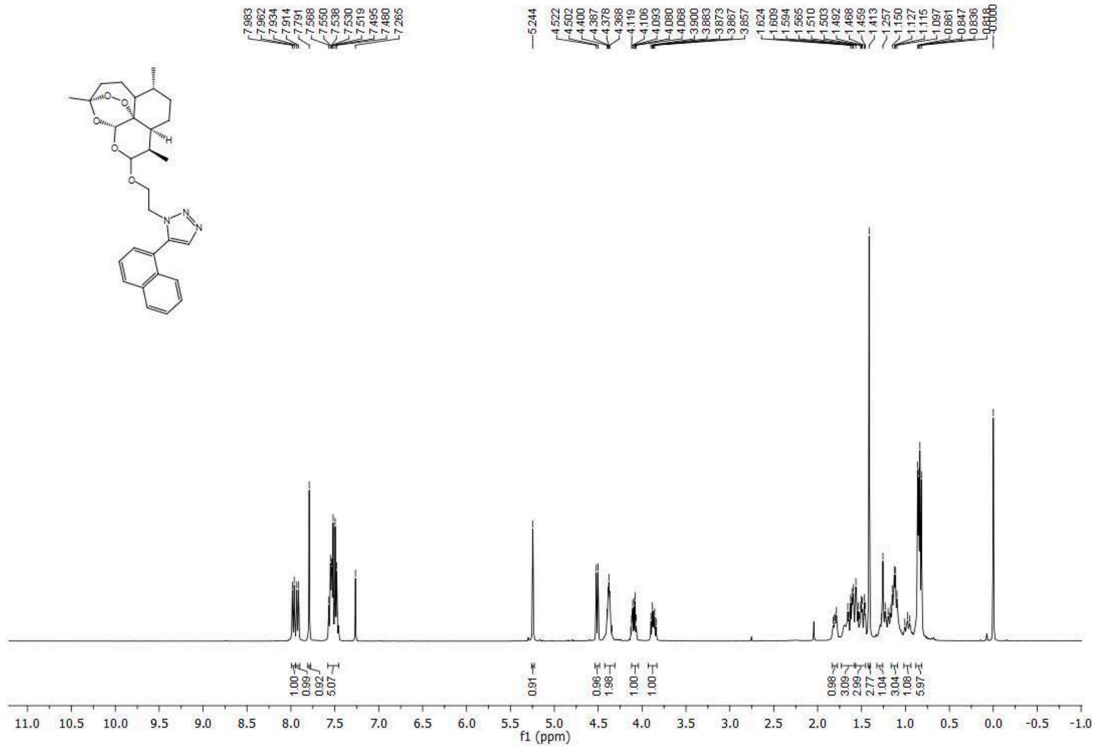

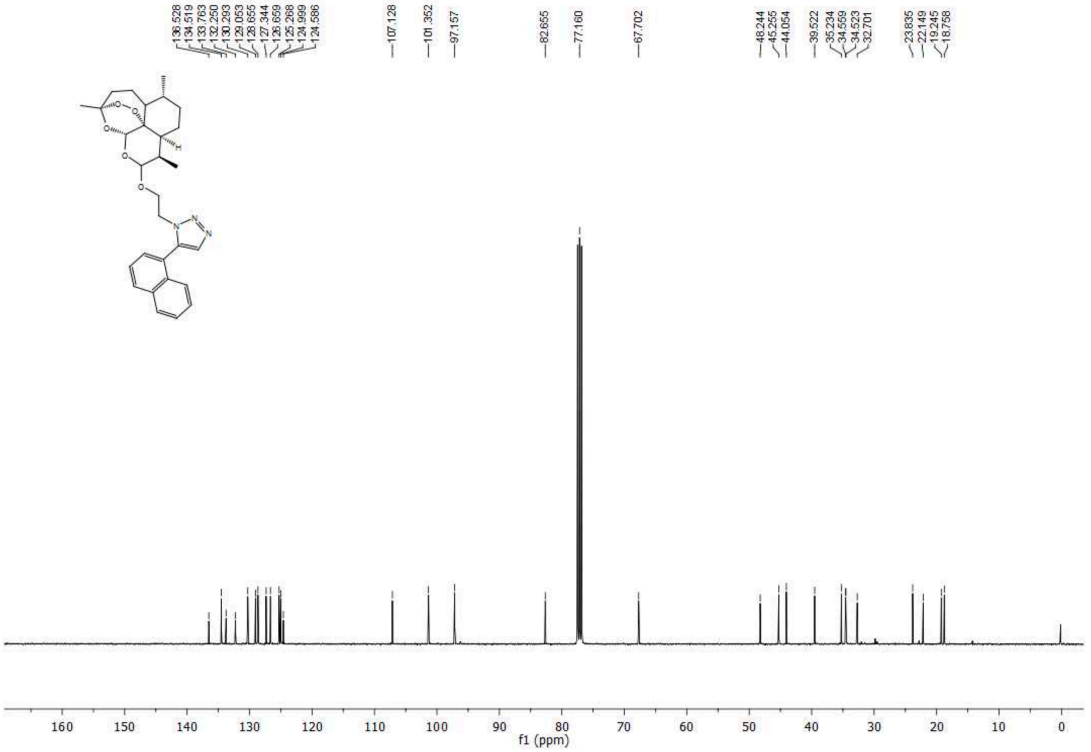

Figure S8. ¹H and ¹³C spectra of **8h**.

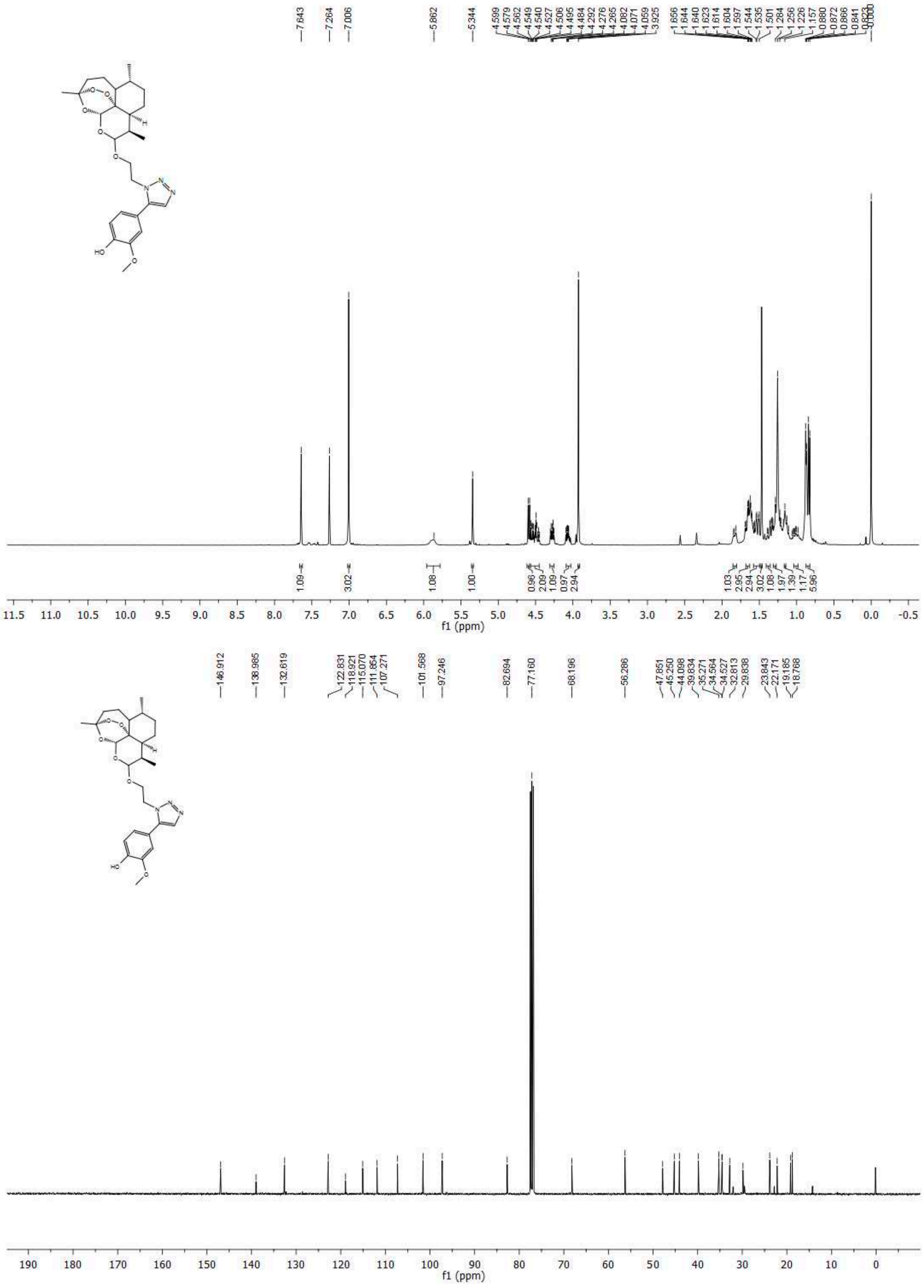

Figure S9. ^{1}H and ^{13}C spectra of **8i**.

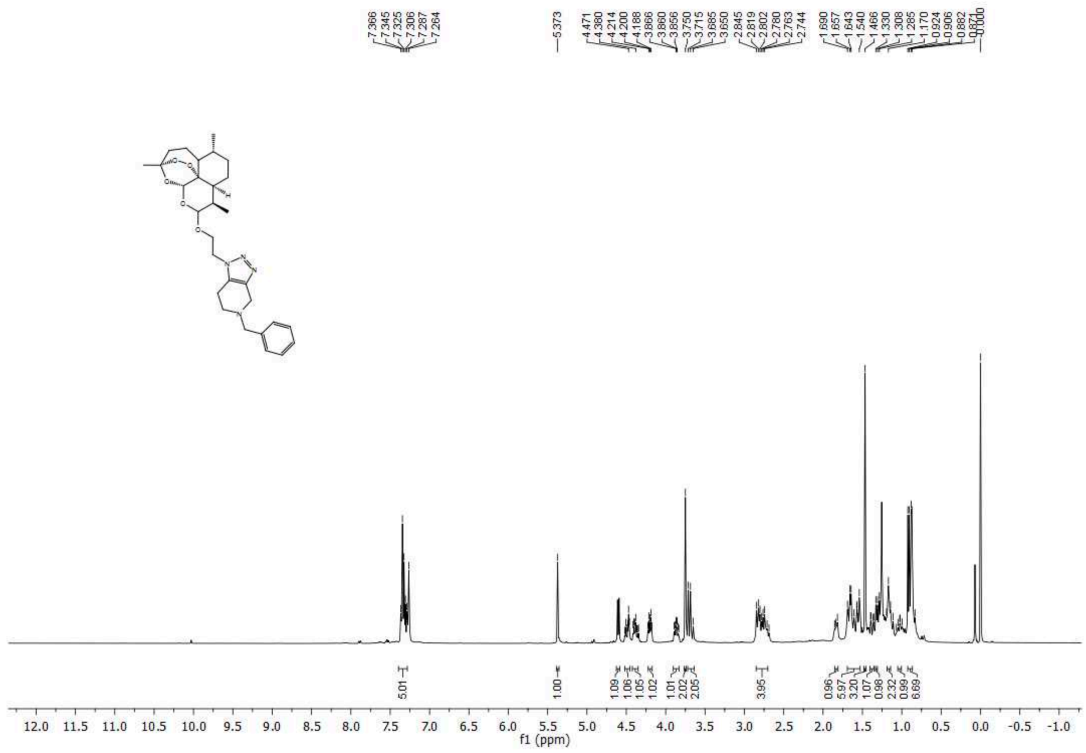

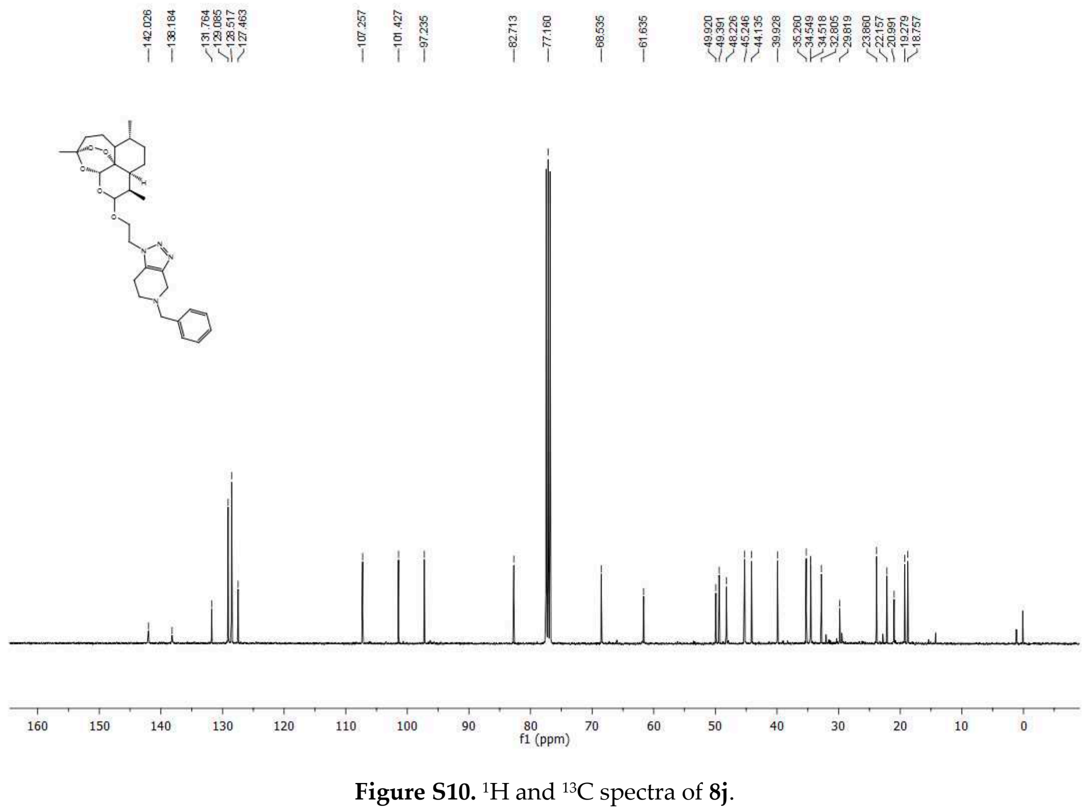

Figure S10. ^{1}H and ^{13}C spectra of **8j**.

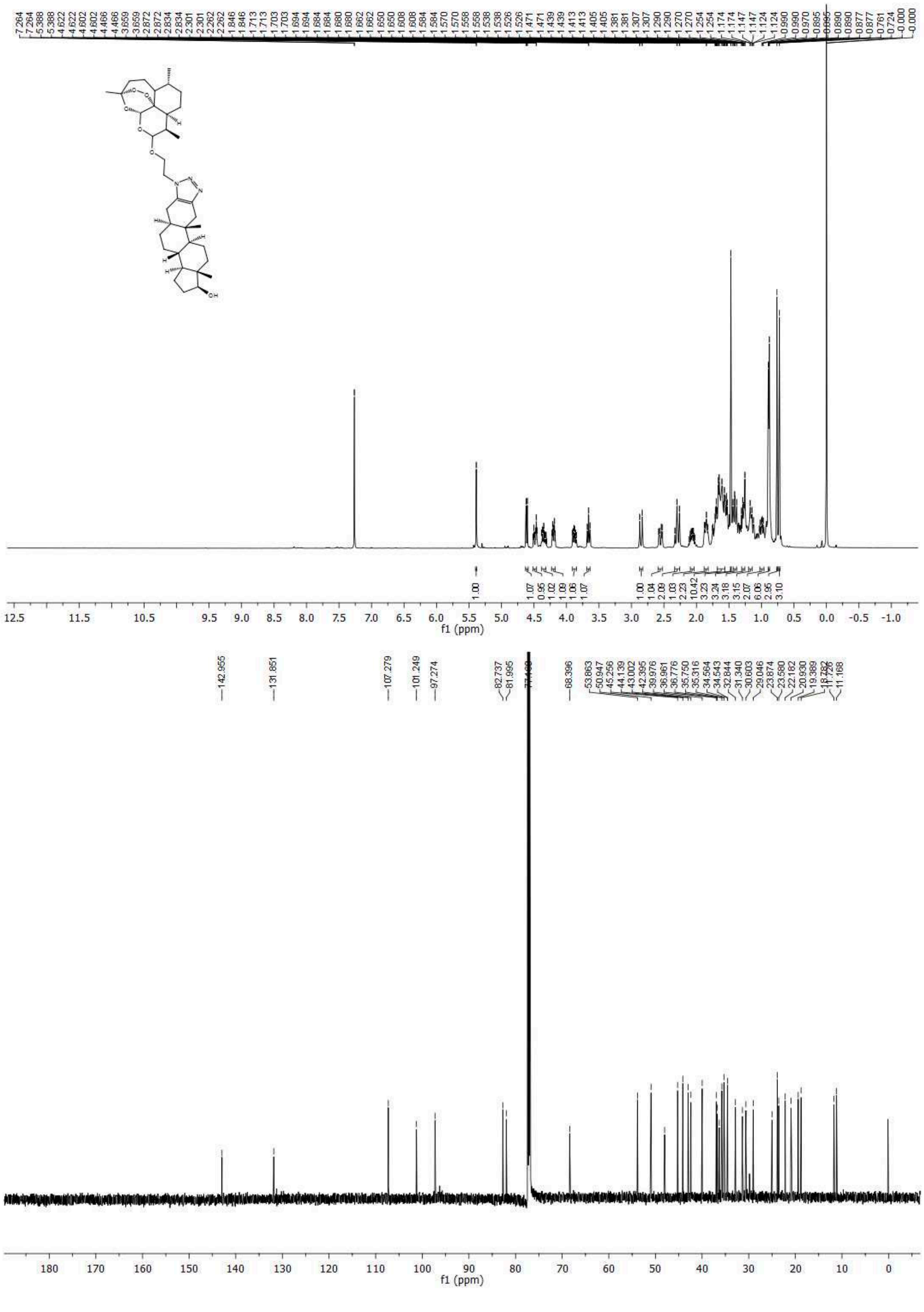

Figure S11. ^{1}H and ^{13}C spectra of **8k**.

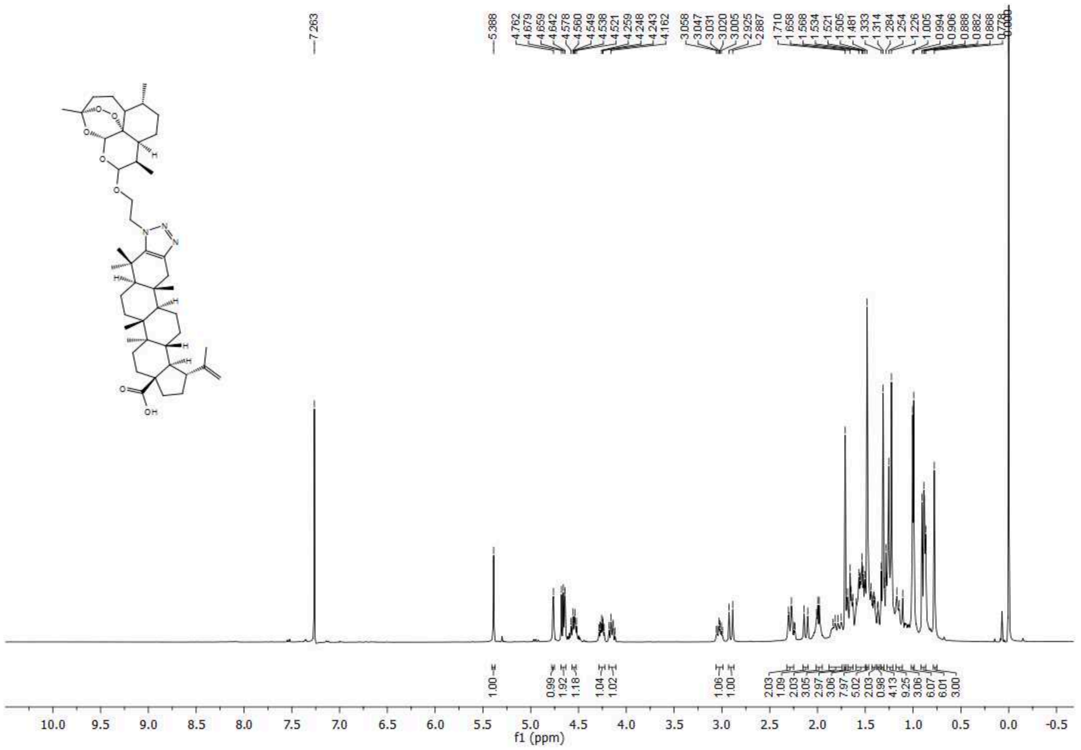

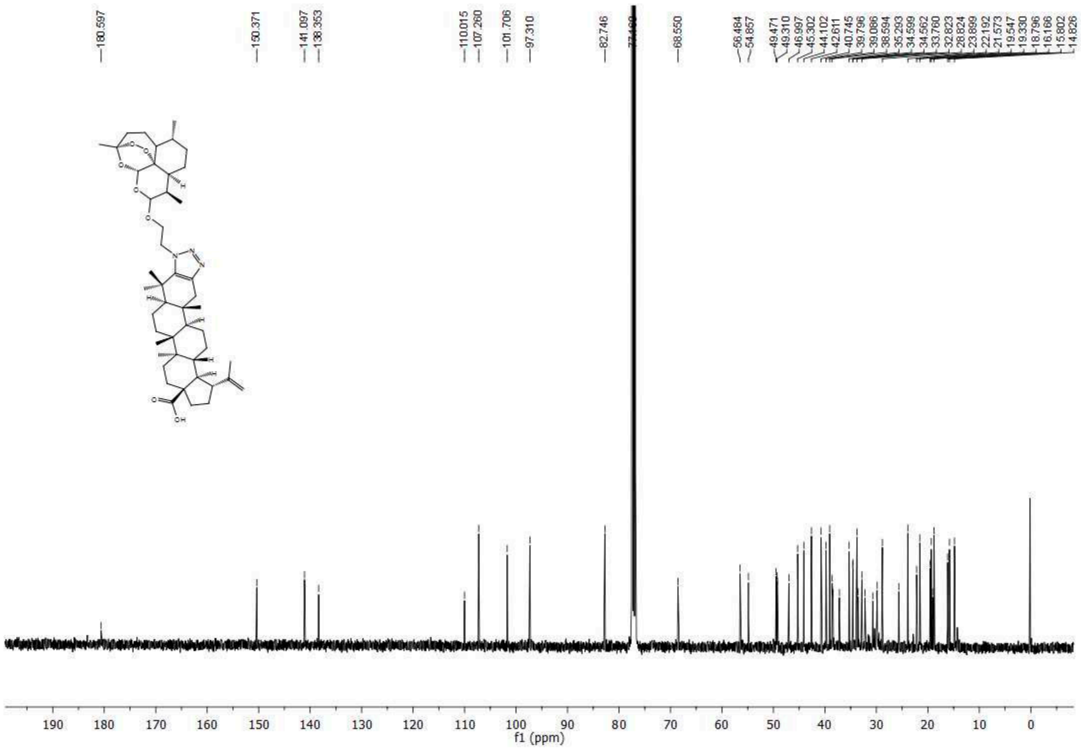

Figure S12. ^{1}H and ^{13}C spectra of **8l**.

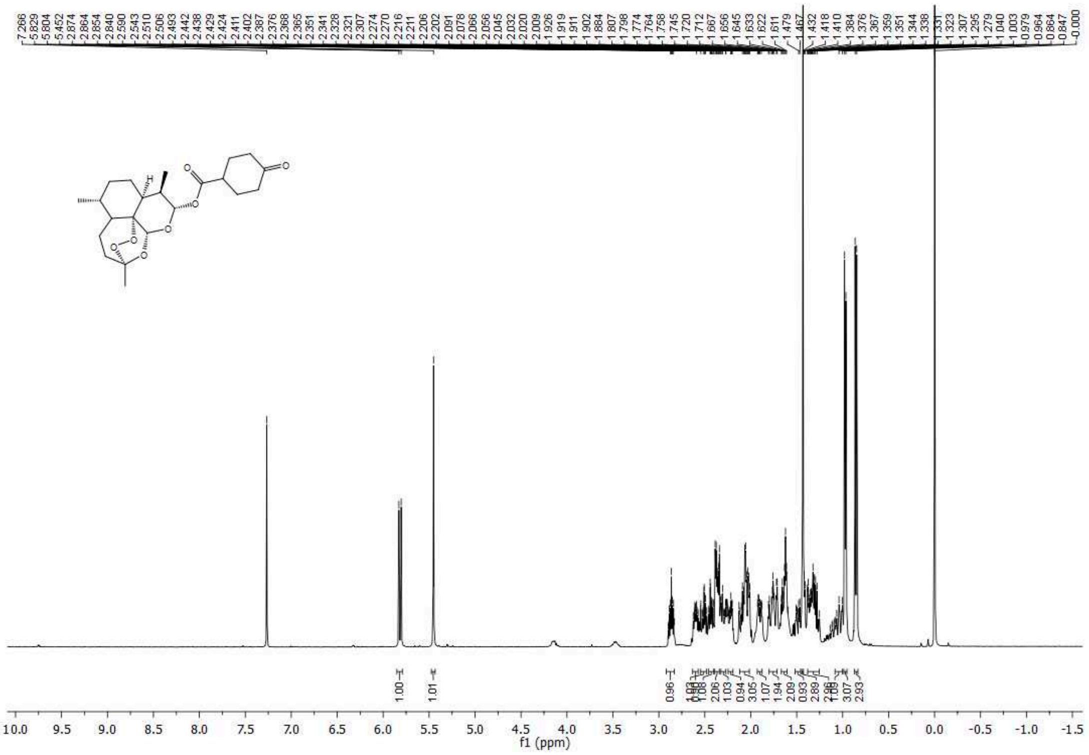

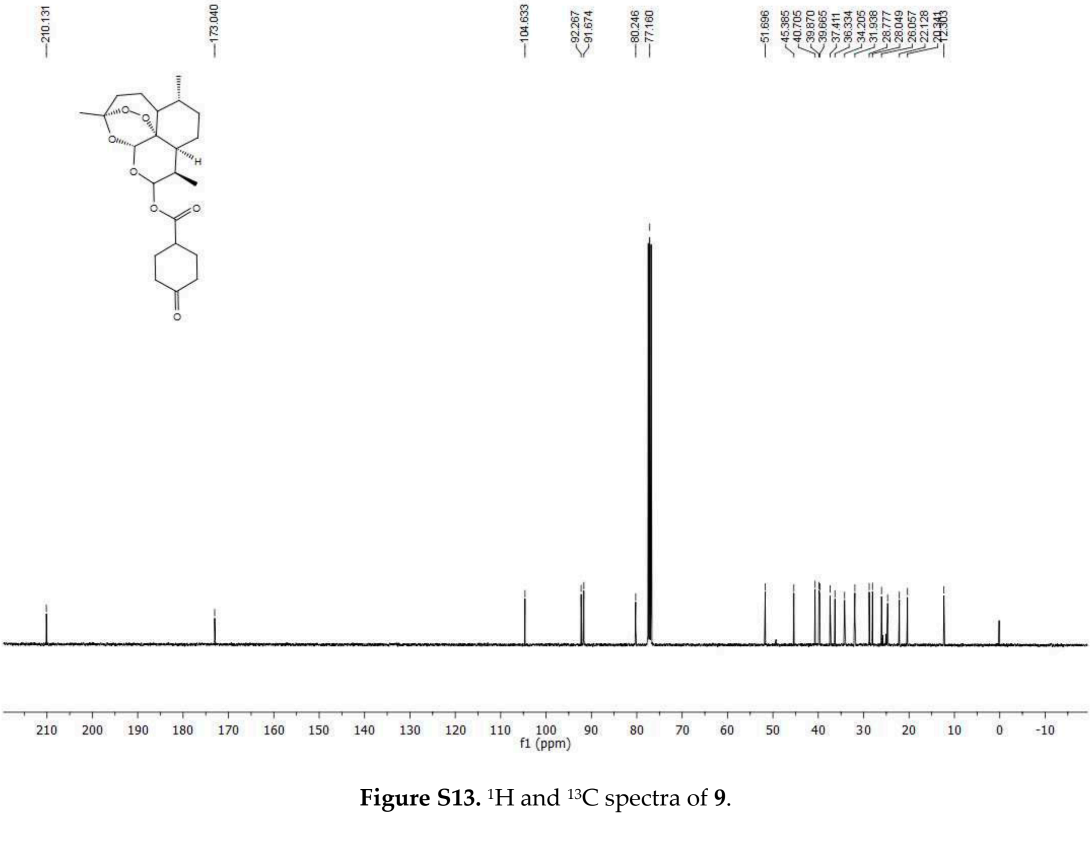

Figure S13. ^{1}H and ^{13}C spectra of **9**.

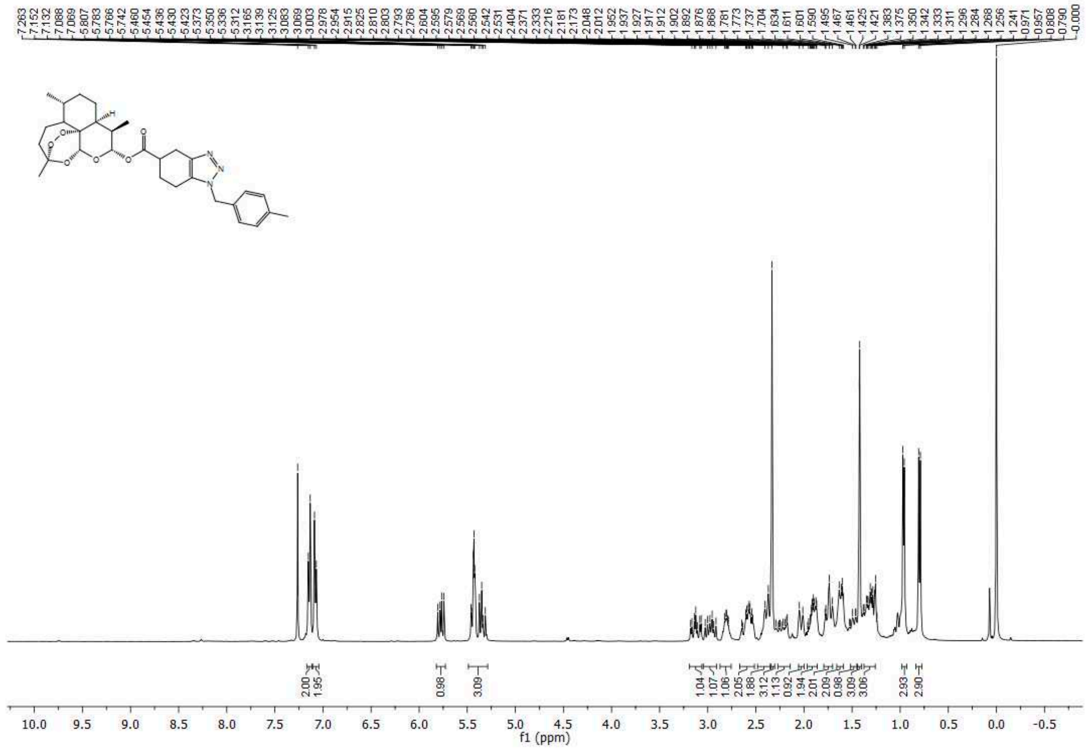

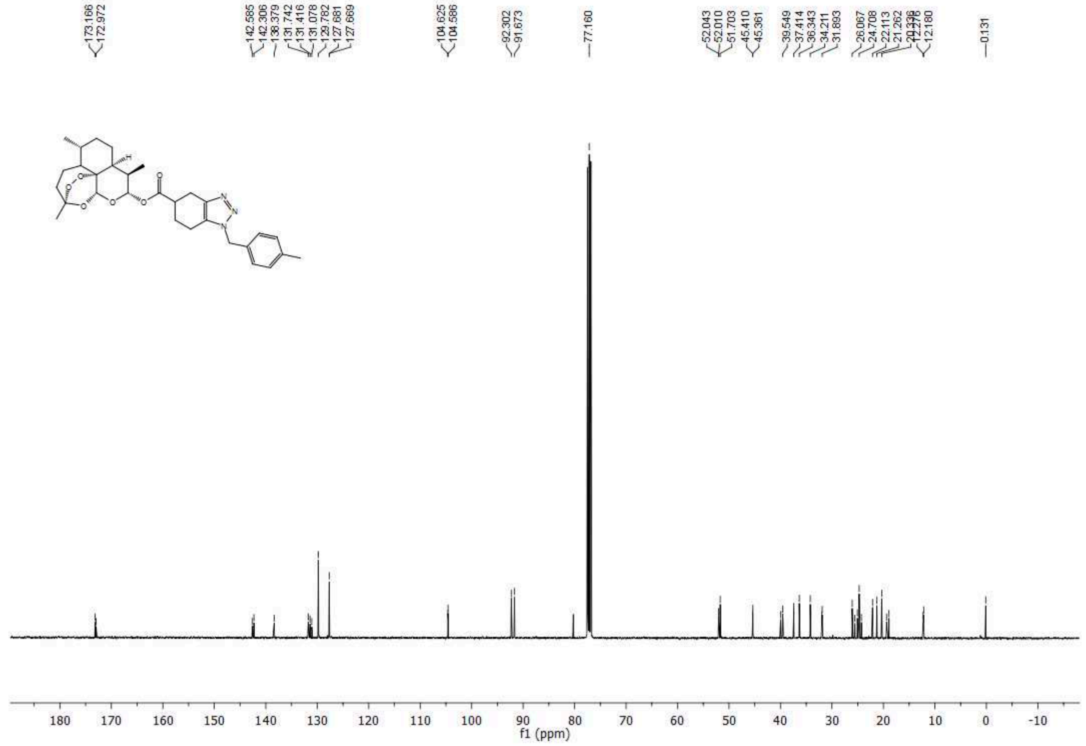

Figure S14. ^{1}H and ^{13}C spectra of **11a**.

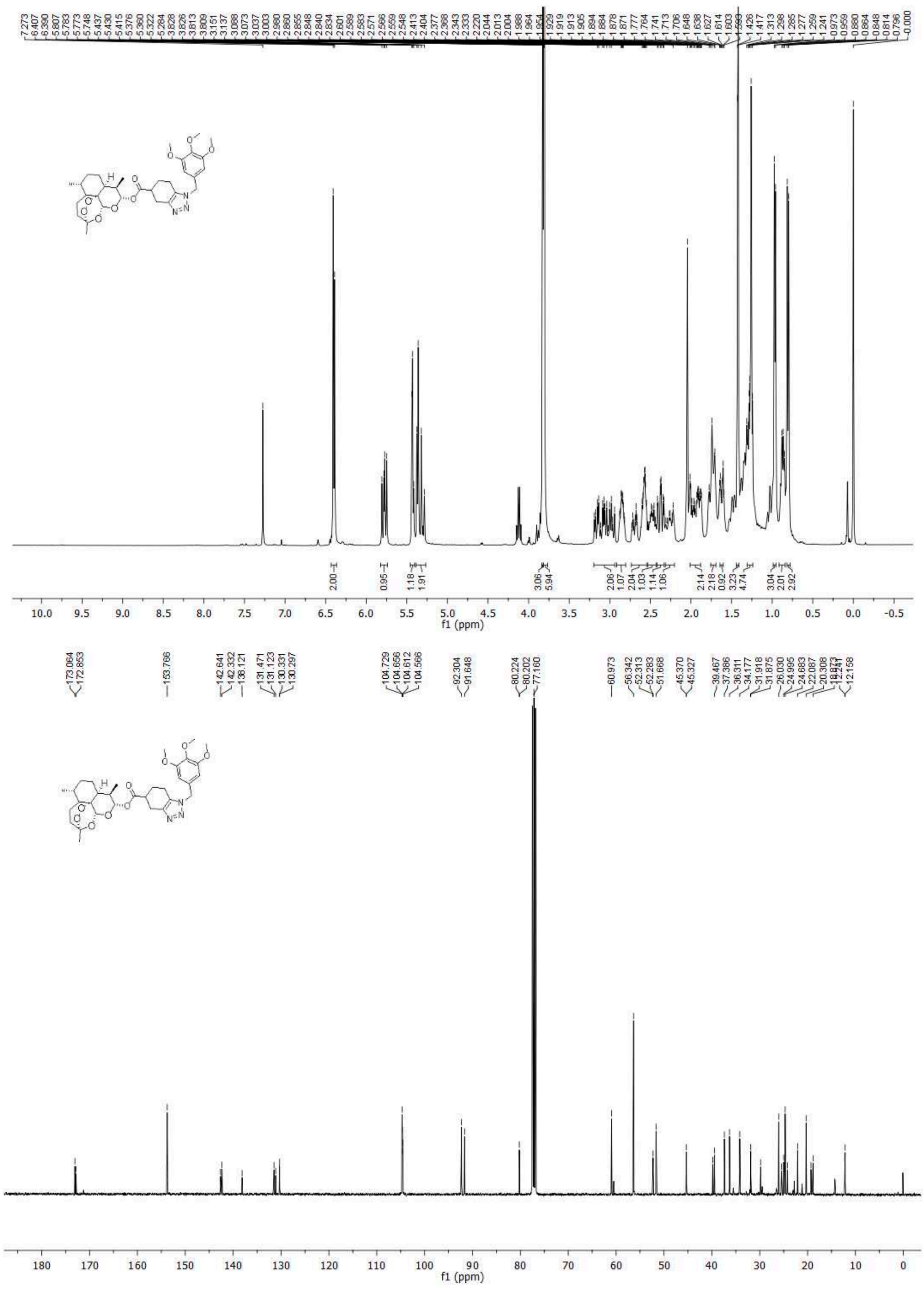

Figure S15. ^{1}H and ^{13}C spectra of **11b**.

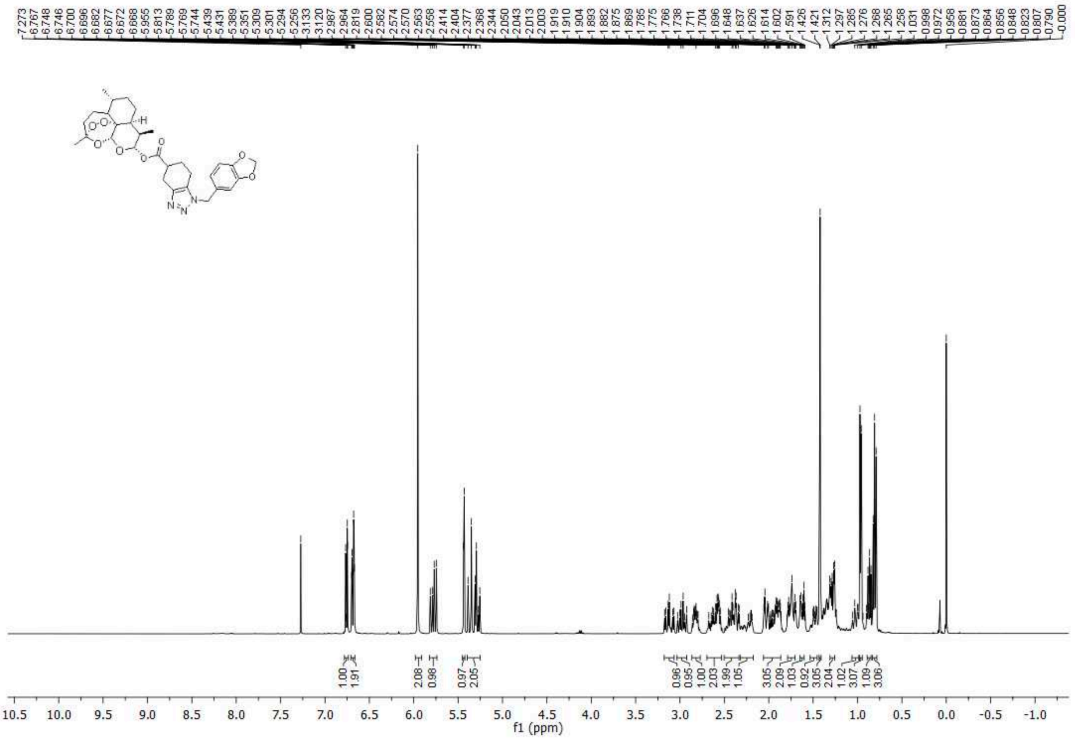

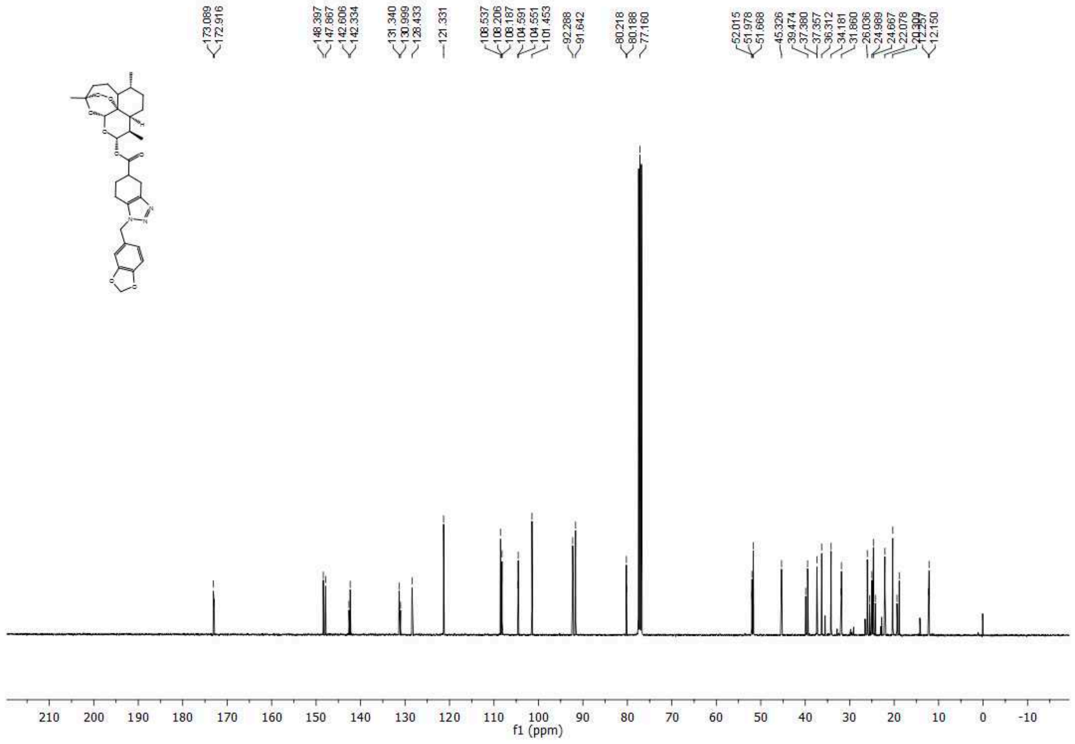

Figure S16. ^{1}H and ^{13}C spectra of **11c**.

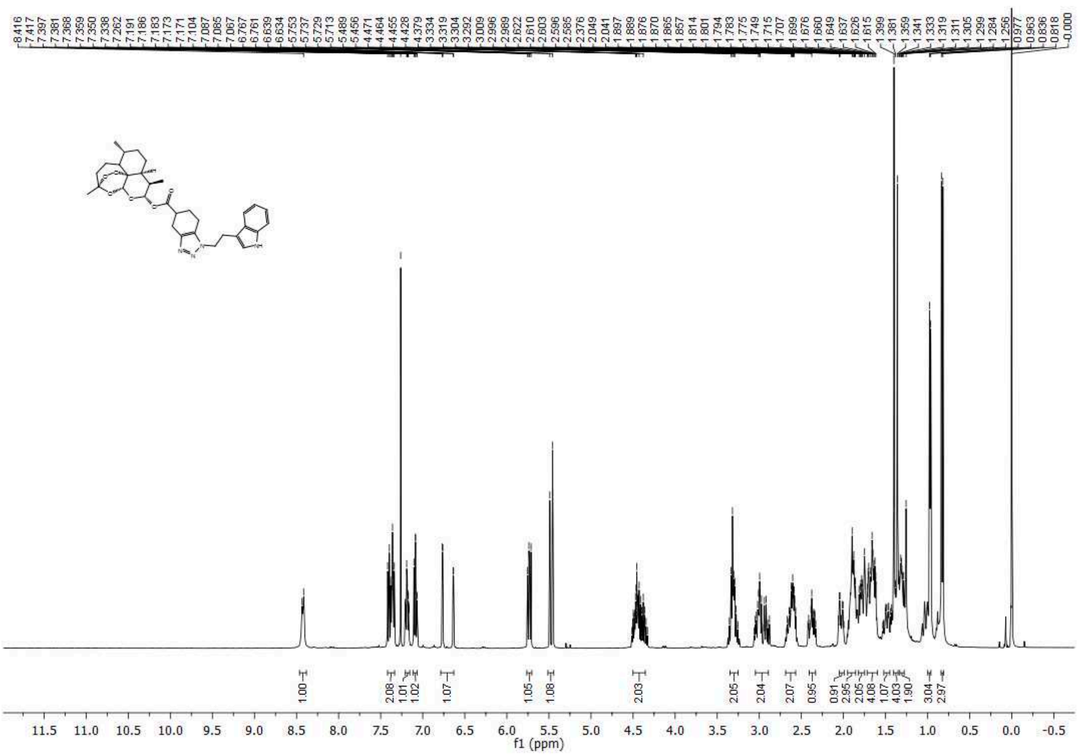

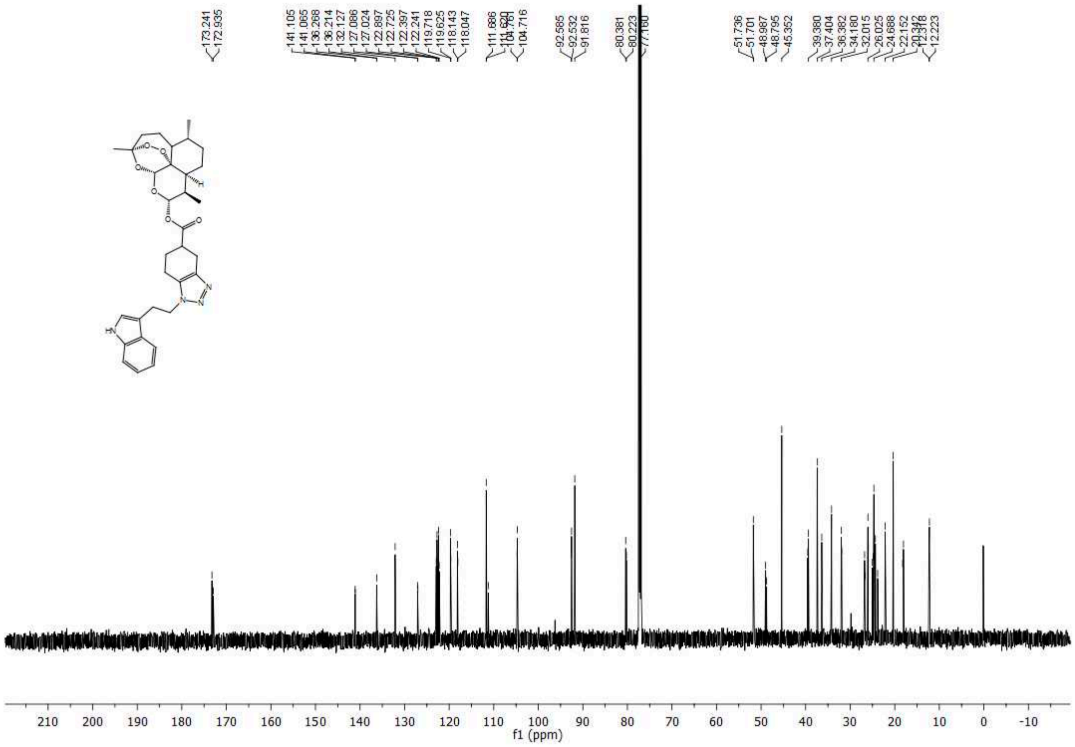

Figure S17. ^{1}H and ^{13}C spectra of **11d**.
